# The impact of B‐cell reconstitution on mRNA vaccine responses in allogeneic stem cell transplant recipients

**DOI:** 10.1002/cti2.70077

**Published:** 2026-02-11

**Authors:** Fredrika Hellgren, Rodrigo Arcoverde Cerveira, Gustaf Lindgren, Puran Chen, Klara Lenart, Sebastian Ols, Alberto Cagigi, Davide Valentini, Mireia Rocavert Barranco, Evangelin Shaloom Vitus, Martin Corcoran, Yong‐Dae Gwon, Mattias NE Forsell, Magnus Evander, COVAXID Study Group, Peter Bergman, Marcus Buggert, Hans‐Gustaf Ljunggren, Soo Aleman, Gunilla B Karlsson Hedestam, Andreas Björklund, Anna Nordlander, Per Ljungman, Stephan Mielke, Karin Loré

**Affiliations:** ^1^ Division of Immunology and Respiratory Medicine, Department of Medicine Solna Karolinska Institutet, Sweden & Karolinska University Hospital Stockholm Sweden; ^2^ Center for Molecular Medicine Karolinska Institutet Stockholm Sweden; ^3^ Department of Cellular Therapy and Allogeneic Stem Cell Transplantation (CAST) Karolinska University Hospital Huddinge Stockholm Sweden; ^4^ Karolinska Comprehensive Cancer Center Karolinska Institutet and University Hospital Stockholm Sweden; ^5^ Karolinska ATMP Center Karolinska Institutet and University Hospital Stockholm Sweden; ^6^ Department of Medicine Huddinge Karolinska Institutet Stockholm Sweden; ^7^ Department of Laboratory Medicine, Biomolecular and Cellular Medicine (BMC) Karolinska Institutet Stockholm Sweden; ^8^ Department of Microbiology, Tumor and Cell Biology Karolinska Institutet Stockholm Sweden; ^9^ Department of Clinical Microbiology Umeå University Umeå Sweden; ^10^ Department of Laboratory Medicine, Clinical Immunology Karolinska Institutet Stockholm Sweden; ^11^ Department of Clinical Immunology and Transfusion Medicine Karolinska University Hospital Stockholm Sweden; ^12^ Department of Infectious Diseases Karolinska University Hospital Stockholm Sweden; ^13^ Present address: Rockefeller University New York NY USA; ^14^ Present address: University of Washington Seattle WA USA; ^15^ Present address: International Vaccine Institute Europe Regional Office Stockholm Sweden

**Keywords:** B cell receptor, CAR T cell, mRNA vaccine, stem cell transplant, transitional B cell

## Abstract

**Objectives:**

Vaccine responses in haematopoietic stem cell transplant (alloHCT) recipients vary, with different degrees of B‐cell reconstitution likely playing a key role. However, mechanistic understanding of the B‐cell receptor (BCR) repertoire and its functional impact post‐alloHCT remain limited.

**Methods:**

Within the scope of a mRNA SARS‐CoV‐2 vaccine phase IV clinical trial in alloHCT recipients (*n* = 77), we have measured antibody titers and avidity, and performed B‐cell immunophenotyping and B‐cell receptor repertoire sequencing in sub‐populations.

**Results:**

AlloHCT patients receiving prime‐boost mRNA vaccination within 12 months post‐transplant exhibited lower vaccine‐specific antibody levels and memory B‐cell frequencies than vaccinated healthy controls. Responses were comparable to healthy controls in patients vaccinated later than 12 months post transplant. BCR repertoire sequencing showed reduced somatic hypermutation (SHM) levels in bulk IgG+ B cells from alloHCT patients. Although some alloHCT patients showed exceptional expansion of a few IgG clones of unknown specificity, their overall B‐cell repertoires remained polyclonal. Vaccine‐specific B‐cell clonotypes detected in patients responding to vaccination showed similar proportional expansion and SHM as in controls. The level of immature CD24hiCD38hi transitional B cells pre‐vaccination was negatively correlated to the vaccine response, and can be used as a predictor of antibody titres.

**Conclusion:**

Our data indicate that mRNA vaccination can stimulate expansion of vaccine‐specific B cells to affinity mature in many alloHCT recipients, though restricted by the presence of immature B‐cell populations.

## Graphical abstract

We characterised B‐cell responses to vaccination in allogeneic haematopoietic stem cell transplant (alloHCT) recipients. While mRNA prime‐boost vaccination stimulated expansion of vaccine‐specific B cells to affinity mature in a majority of alloHCT recipients, responses were variable and seemingly restricted by the presence of immature B‐cell populations.

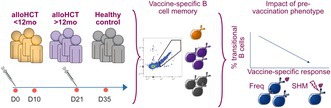

## Introduction

Allogeneic haematopoietic stem cell transplantation (alloHCT) is a well‐established standard‐of‐care cellular immunotherapy for patients with haematological and immune disorders.^1^ While alloHCT is a life‐saving intervention, transplant recipients have an increased vulnerability to infections during the initial period of immunodeficiency post‐transplant because of pre‐graft conditioning and post‐graft immunosuppression. Patients are at higher risk of developing infection‐related complications in the vulnerable phase in the first 12 months following alloHCT. This period of vulnerability extends further if immune suppression must be prolonged or re‐initiated because of the appearance of acute and chronic GvHD (cGvHD). Although reimmunisation of alloHCT recipients with childhood vaccines is broadly recommended, immune responses to vaccination remain heterogeneous, and protection is not always reliably achieved when using standard vaccine regimens.^2^ The improvement of vaccination strategies in alloHCT recipients is subject to ongoing investigation and requires a more complete understanding of the reconstituted immune repertoire post‐alloHCT.

We have previously reported primary endpoint data of a prospective phase IV vaccine trial in immunocompromised patients, including recipients of alloHCT or B cell‐directed CAR‐T cell therapy.^3^ Besides documenting safety and efficacy, we identified that time elapsed post‐transplantation and the presence of severe chronic GvHD impacted seroconversion of alloHCT recipients, while CAR‐T recipients had no detectable seroconversion.^3^ Concordantly, improved vaccine responses with increasing time elapsed from alloHCT to vaccination have previously been demonstrated for both influenza and SARS‐CoV‐2 vaccination; however, subsets of alloHCT recipients remain seronegative after vaccination, despite the administration of booster doses.^4^, ^5^ In accordance with the observations from our clinical trial,^3^ chronic GVHD, B cell aplasia^6^, ^7^, ^8^ and ongoing immunosuppression^8^, ^9^ are among the factors that have been associated with insufficient immune responses to vaccines.^6^, ^10^

In contrast to NK and T‐cell immune reconstitution, B‐cell reconstitution is usually delayed after alloHCT, with donor‐derived transitional B cells (TrB) dominating the early phases but diminishing over time and giving rise to mature B cells in the later phases.^11^, ^12^, ^13^, ^14^, ^15^ The early post‐alloHCT B cell pool has been shown to contain high proportions of immature transitional B cells, with gradual progression to normal proportions of mature naïve B cells.^11^, ^16^ The composition of the B‐cell pool likely affects responsiveness to vaccination.^10^ However, vaccine responses post‐alloHCT have mainly been evaluated by quantification of serum antibodies, which do not reflect the memory B‐cell repertoire. The human BCR repertoire is highly diverse,^17^ albeit with evidence of shared clonal features across individuals.^18^ The composition of the BCR repertoire and the frequency of antigen‐reactive precursors can influence immune responses to vaccination.^19^, ^20^, ^21^ We have previously shown that the diversity of the antigen‐specific BCR repertoire is associated with the breadth of virus neutralisation.^22^ However, the influence of B‐cell phenotype, clonal composition and BCR diversity on vaccine‐induced immune responses in alloHCT recipients is not known. The few existing studies describing BCR repertoires after alloHCT have shown oligoclonality of the B‐cell pool early after transplantation,^16^, ^23^ as well as reduced somatic hypermutation (SHM)^24^ persisting for at least 1 year post alloHCT. In this study, we performed a prospective in‐depth analysis of post‐alloHCT B‐cell responses to vaccination and evaluated the association to B‐cell reconstitution at the immunophenotype and BCR sequence levels.

## Results

### AlloHCT recipients develop functional antibody responses to two‐dose mRNA vaccination while CAR T cell treated patients were unable to respond

**Figure 1:**
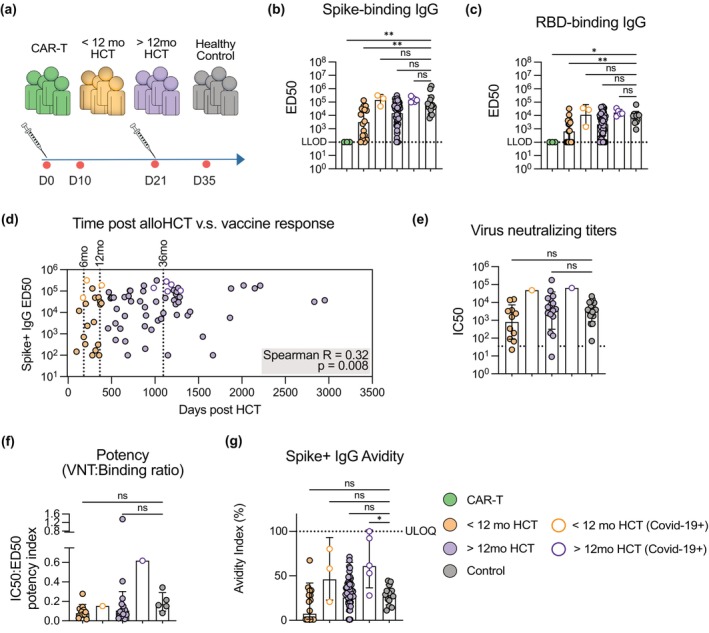
**(a)** Overview of study groups. **(b)** Serum spike‐binding IgG titres measured by ELISA. Reported as ED50. **(c)** Serum RBD‐binding IgG titres measured by ELISA. Reported as ED50. **(d)** Nonparametric Spearman correlation of serum spike‐binding IgG and time elapsed since alloHCT. AlloHCT recipients with pre‐existing immunity and/or known COVID‐19 infection are shown in the plot but excluded from correlation analysis. **(e)** Live virus neutralising serum titres against Wu‐Hu‐1 strain of SARS‐CoV‐2, quantified as IC50. **(f)** Neutralisation potency index, defined as the ratio of virus neutralisation (IC50) to binding titretitre (ED50) measured by ELISA. **(g)** Binding avidity of Spike+ IgG in serum measured by chaotropic ELISA using 1.5 m NaSCN. Avidity index calculated as % of Spike‐binding IgG detectable after NaSCN treatment relative to PBS‐treated parallel control. Samples below detection threshold after NaSCN treatment reported as index = 1%. Bar and error indicate geometric mean ± geometric SD. *P‐value < 0.05, **P‐value < 0.01.

AlloHCT recipients (*n* = 77), CAR‐T recipients (*n* = 3) or healthy adults (*n* = 29) were vaccinated with two doses of the BNT162b2/Comirnaty® mRNA vaccine 3 weeks apart (Figure 1a).^3^ The study cohort characteristics at study start are summarised in Table 1 and Supplementary table 1 (S2). Data types across study participants are reported in Supplementary table 1. Eight alloHCT recipients were grouped as COVID‐19‐experienced on the basis of detectable antibodies for SARS‐CoV‐2 at study start (*n* = 7) and/or PCR‐verified infection before study day 35 (*n* = 2, one also with pre‐existing antibody). These study participants were grouped as COVID‐19‐experienced and reported separately in Figure 1.

**Table 1:** Summary of cohort characteristics

Key data types (Figure reference)	Full alloHCT/CAR‐T population (any analysis)		Repertoire sequencing alloHCT		Full HC population (any analysis)		Repertoire sequencing HC	
Type of treatment								
AlloHCT	77		16		29		4	
CAR T	3		0					
Time from transplantation								
< 12 months	21	27%	7	44%				
> 12 months	56	73%	9	56%				
Sex								
Male	43	54%	6	38%	14	48%	3	75%
Female	37	46%	10	63%	15	51%	1	25%
Age								
Median; min–max	59; 19–74		51.5; 20–74		55; 22–79		41;22–52	
Diagnosis								
AML	21		5					
ALL	10		3					
MDS	19		4					
MPN	4		0					
Lymphoma	17		3					
Myeloma	3		1					
Non‐malignant	5		0					
CML	1		0					
Donor type								
HLA‐identical sibling	22		5					
Haploidentical family	3		1					
Unrelated donor	52		10					
Autologous/CAR T	3		0					
Stem cell source								
Bone marrow	4		2					
Peripheral blood	73		14					
Conditioning intensity								
Myeloablative	13		6					
Reduced intensity	64		10					
T cell depletion								
ATG	58		10					
Post‐Cy	3		1					
None	16		5					
Ongoing immunosuppression								
Yes	22	29%	7	44%				
No	55	71%	9	56%				
Prevaccination seropositive	7^a^		1^b^					
COVID‐19 before day + 35	2		0					

Full summary of characteristics for specific key subsets of data can be found in Supplementary table 1 (S2).

^a^ One both seropositive and PCR positive.

^b^ Excluded from vaccine‐specific repertoire analysis.

At 14 days after boost vaccination, there were detectable IgG binding titres against both spike (S) and the receptor binding domain (RBD) in all healthy controls assessed (*n* = 14), as well as 69/77 (90%) of alloHCT recipients for S, and 57/77 (74%) for RBD. No detectable S‐ or RBD‐specific IgG titres were found in recipients of B cell targeted CAR‐T therapy (Figure 1b and c). AlloHCT recipients vaccinated < 12 months (± 30 days) post‐transplant mounted lower vaccine‐specific antibody levels than healthy controls, while antibody responses in patients vaccinated later than 12 months post transplant were comparable to healthy controls (Figure 1b and c). Low‐ or undetectable S‐/RBD‐specific titres were noted in both HCT groups. S‐specific IgG antibody levels significantly correlated with time elapsed between alloHCT and first vaccine dose (Figure 1d). However, the correlation was limited and outliers observed, indicating that factors other than time also influence the magnitude of the vaccine response. Further subdivision of the > 12mo HCT group did not indicate that a different time cut‐off (within a 12–36 months range) would consistently separate responders from non‐responders (Supplementary figure 2A).

Stratification of alloHCT recipients by ongoing pharmacologic immunosuppression (Supplementary figure 3A) and cGvHD at the time of vaccination (Supplementary figure 3D) indicated that both variables negatively impacted the magnitude of Spike‐specific IgG in serum, though significance was only seen in alloHCT recipients < 12mo post alloHCT.

S‐specific memory T cells, as assessed by peptide recall and intracellular cytokine staining, showed detectable CD4^+^ and CD8^+^ T‐cell responses in a majority of the participants assessed, including the CAR‐T recipients (Supplementary figure 4A and B). The S‐specific T‐cell response was Th1 polarised as evidenced by the majority of responding cells producing IFN‐γ or IL‐2, as well as TNF.

### Qualitatively similar vaccine antibody responses in seroconverted alloHCT recipients and healthy controls

Serum neutralising capacity against live SARS‐CoV‐2 (WuHu‐1 strain) after two doses of mRNA vaccine was assessed in a subset of alloHCT recipients (*n* = 29) and healthy controls (*n* = 19). Selection was based on both sample availability and demonstrated seroconversion by S‐ and RBD‐binding ELISA. Seroconverted alloHCT recipients demonstrated similar neutralising titres as the healthy controls (Figure 1e),  as well as neutralisation potency index (ratio of neutralising titre to binding ELISA titre) (Figure 1f). An assessment of antibody binding strength by chaotropic ELISA showed on average similar binding strength in alloHCT recipients and healthy controls (Figure 1g). We observed a trend towards a markedly higher Spike‐binding IgG avidity in SARS‐CoV‐2 infection‐experienced participants, in line with prior data from healthy vaccinees.^25^ A proportion of alloHCT recipients had antibody avidity below quantifiable level (no detectable binding remaining after chaotropic treatment, here reported as avidity index = 1%), which was not seen in the controls. Assessment of antibody cross‐reactivity to the S proteins of the gamma and beta strains of SARS‐CoV‐2 showed similar levels in vaccine‐responsive alloHCT recipients and healthy controls (Supplementary figure 3G). The induction of antibodies targeting different regions of the S/RBD protein was investigated by performing ELISA with the competition of well‐characterised monoclonal antibodies (mAbs) representing four previously defined antibody classes^26^ for a subset of participants. Among alloHCT recipients with measurable antibody levels allowing for accurate quantitation, recipients and healthy controls exhibited similar levels of mAb‐competing binding for all five mAbs analysed (Supplementary figure 3H). In summary, in the alloHCT recipients who developed quantifiable antibody responses to mRNA vaccination, antibodies exhibited similar avidity, cross‐reactivity and neutralising capacity as in a healthy population. However, the underlying reasons for the observed highly variable vaccine responses in the alloHCT patients remained unclear.

### Clonality and SHM of the IgG B‐cell repertoire is altered in alloHCT recipients compared to healthy controls

To better understand how immune reconstitution and the composition of the B‐cell pool may impact the vaccine response, we performed IgM and IgG repertoire sequencing of the BCR immunoglobulin heavy chain variable region (VH) for bulk B cells from 20 individuals representing both early (< 12) and late (> 12) months post alloHCT recipients as well as healthy controls. Given that alloHCT recipients constitute such a highly heterogeneous group, we selected both individuals with (*n* = 8) and without (*n* = 8) GvHD for this analysis. Detailed sequencing information per group is described in Supplementary Table 2.

**Figure 2:**
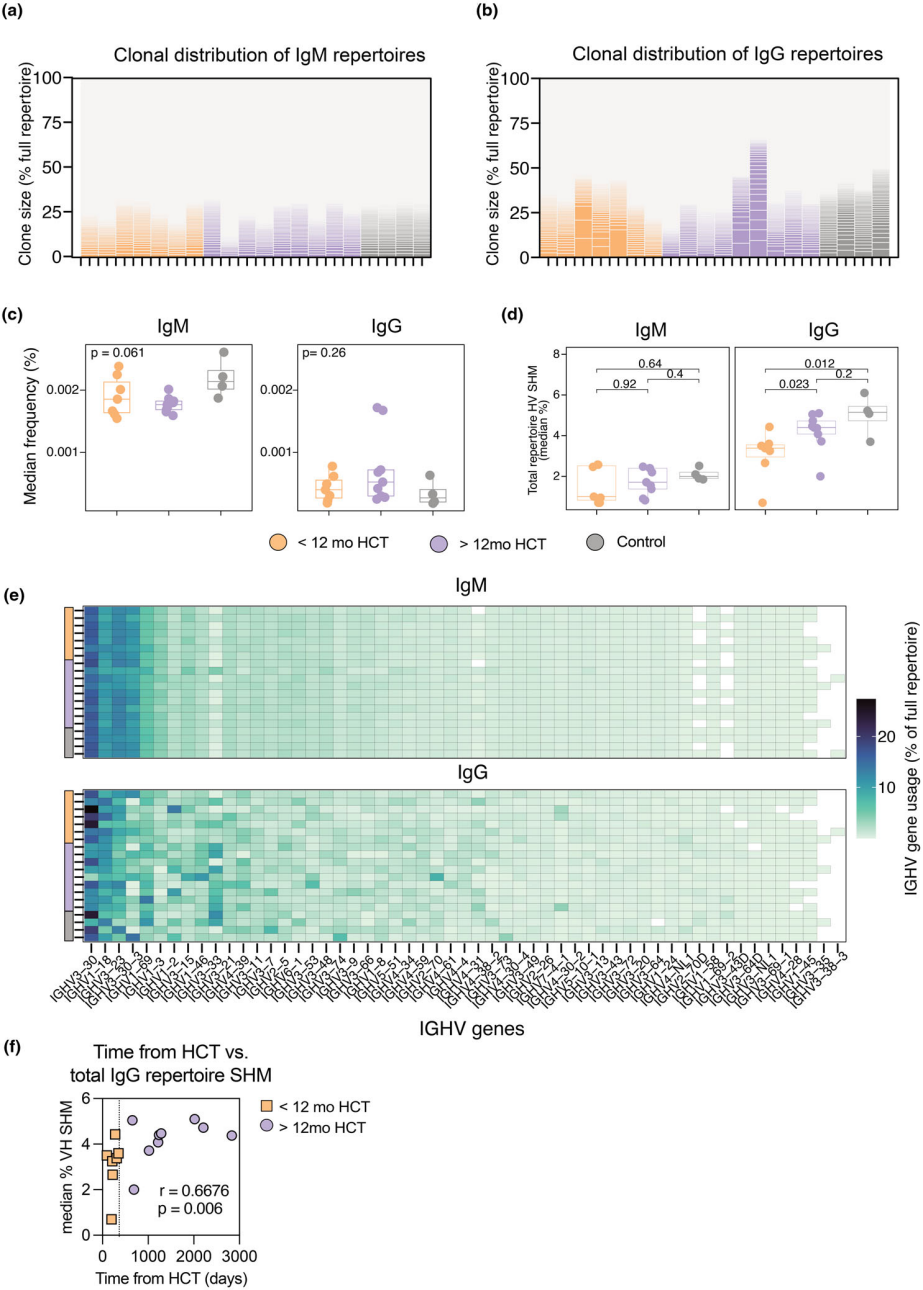
**(a, b)** Proportional distribution of IgM **(a)** and IgG **(b)** BCR clonotypes identified per repertoire sequenced. Each column represents one repertoire and study subject. Each line/block represents one clone. Clonotypes are defined by identical heavy chain V‐J gene pairing and 80% CDRH3 identity. *Y* axis shows each clonotype as a percentage of the total number of clonotypes identified per repertoire and isotype. **(c)** Median clonotype percentage per individual study participant sequenced. **(d)** Percentage IGHV somatic hypermutation (SHM) accumulated, quantified through alignment to the OGRDB immunoglobulin germline database and SHM calculation using IgDiscover software. Mean clonotype IGHV SHM per individual study participant is shown. **(e)** IGHV gene usage per clonotype and repertoire sequenced. Each row represents one study participant repertoire. Colour scale denotes percentage of clonotypes in total repertoire aligned to a particular IGHV family. **(f)** Nonparametric Spearman correlation of mean clonotype IGHV SHM per study participant vs. number of days elapsed post alloHCT. *N* = 16 (healthy controls not shown in correlation analysis). Sequencing data analysed using IgDiscover version 0.15.1 and in‐house R scripts. *N* = 20 unless otherwise specified. Box plots indicate median ± IQR.

Clonotype analysis was performed with the IgDiscover software, using the well‐established criteria of identical V‐J pairing, same HCDR3 length and 80% amino acid sequence identity in the HCDR3. Both IgM and IgG repertoires were analysed (Figure 2a and b). The IgG repertoires showed variable clonal composition between alloHCT recipients, with some participants exhibiting proportionally expanded clones comprising > 5% (up to approximately 15%) of the total repertoire. Such proportionally expanded IgG clones were not observed in healthy controls. Despite the significantly expanded BCR clones in some alloHCT recipients, median clonal proportions were similar between alloHCT recipients and healthy controls in both IgG and IgM repertoires (Figure 2c). Distribution of VH and JH gene usage in IgG and IgM repertoires was similar between alloHCT recipients and controls (Figure 2e, Supplementary figure 5C). The overall polyclonal repertoires were further supported by the estimation of IgG and IgM BCR clonotype diversity using species richness index Chao1, which yielded similar results in the alloHCT recipients and controls irrespective of time elapsed after transplantation (Supplementary figure 5D).

We estimated the degree of IGHV somatic hypermutation (SHM) compared with germlines assigned using the OGRDB AIRR‐C database (version 7) as reference. Post‐alloHCT repertoires showed a similar median SHM for IgM but reduced SHM of IgG repertoires in the < 12mo group compared with controls (Figure 2d). We also observed that IgG SHM correlated with time post‐alloHCT (Figure 2f), indicating time‐dependent maturation of the class‐switched B‐cell pool. Subdividing alloHCT recipients by the presence/absence of cGvHD did not show a clear effect on IGHV‐SHM (Supplementary figure 7B) or clonal size (Supplementary figure 7C) for either IgM or IgG repertoires.

### Lower frequency but similar SHM and clonality in vaccine‐specific memory B cells of alloHCT recipients

**Figure 3:**
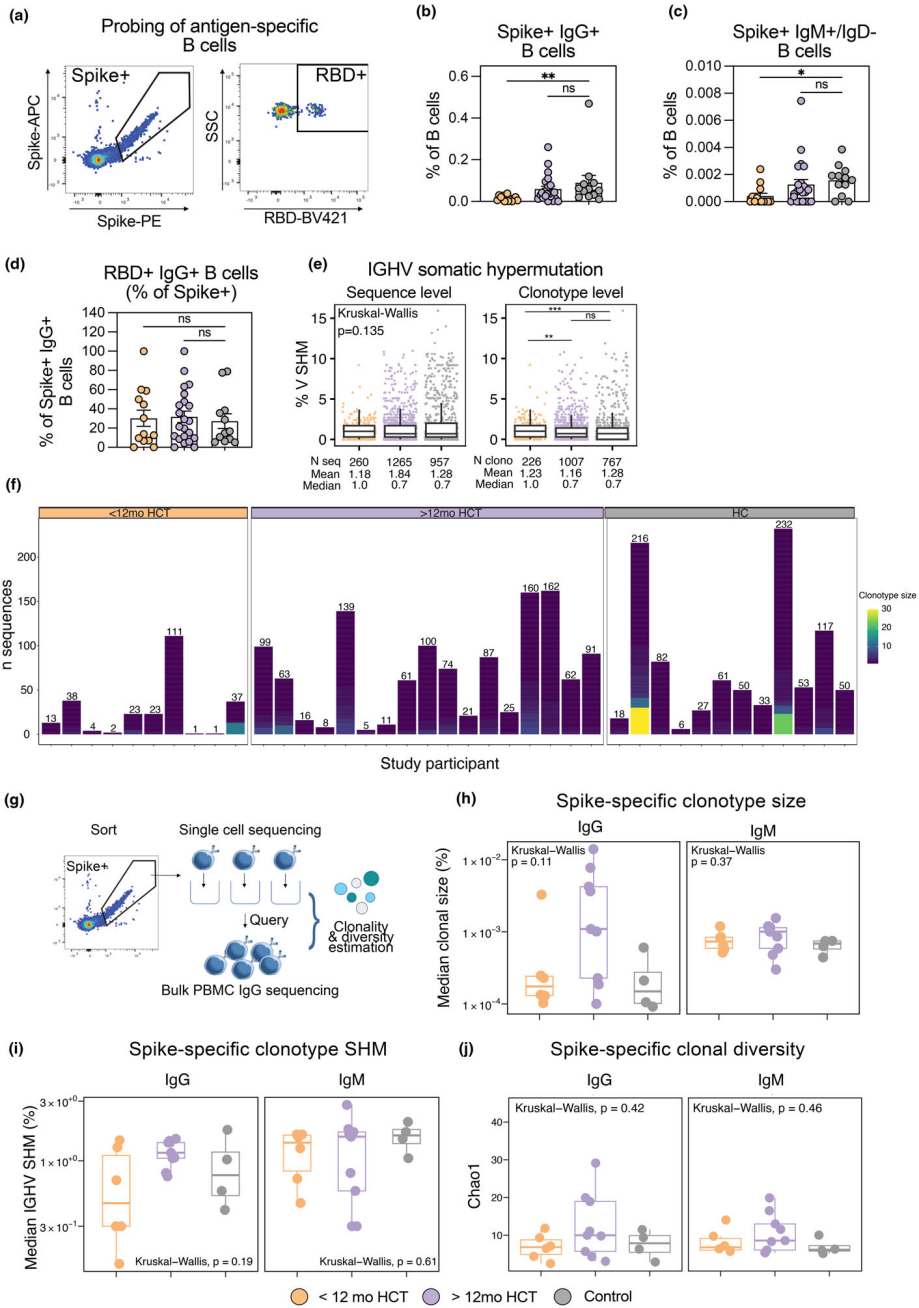
**(a)** Fluorescent probing, gating and sorting strategy for identification of Spike‐ and RBD‐specific B cells. **(b, c)** Enumeration of Spike‐ and RBD‐specific IgG+ (B) and IgM+/IgD‐ (C) B cells. **(d)** Fraction RBD‐binding IgG+ B cells out of total Spike‐binding. **(e)** Box plots showing percentage IGHV somatic hypermutation (SHM) of sorted Spike+ BCR sequences. **(f)** Clonotype distribution of Spike+ MBC IGH sequences. Clonotype definition = identical V‐J pairing and > 80% identity in HCDR3. Each column shows clonotypes for one study participant. Number annotations denote total number of IGH sequences obtained per study participant. **(g)** Illustration of combined bulk and query sequencing analysis. H‐I: Spike‐specific clonotype size **(h)** and IGHV SHM **(i)** using IgDiscover clonoquery to detect likely Spike+ IgG and IgM sequences in bulk repertoire sequencing data, using sequences obtained from fluorescent probe index sorting as query reference database. Data points indicate computed median clonal size and SHM per queried repertoire. **(j)** Assessment of Spike+ clonotype diversity. Datapoints indicate computed Chao1 index per queried repertoire. BCR sequences were aligned to the OGRDB immunoglobulin germline database using IgDiscover software. SHM estimations, clonotyping and clonotype query analyses were performed using IgDiscover. *N* = 47 (Figure 5a–d, 35 alloHCT, 12 HC). *N* = 39 (Figure 5e–h) (27 alloHCT, 12 HC). *N* = 19 (Figure 3i and j) (15 alloHCT, 4 HC). Error bars of bar plots **(b–d)** indicate mean +/− SEM. Lines of box plots indicate median ± IQR. One repertoire shown in Figure 2 (alloHCT group) was not analysed for Spike‐specific clonotypes because of pre‐existing immunity. *P‐value < 0.05, **P‐value < 0.01, ***P‐value < 0.001.

Given the differences in the overall BCR repertoire between alloHCT recipients and healthy controls, we investigated whether differences would also be observed in the antigen‐specific B cells induced by vaccination. The frequencies of S‐ and RBD‐specific memory B cells (MBCs) were measured using fluorescently tagged recombinant S and RBD protein probes (Figure 3a). As with the antibody titres, the frequency of both S‐specific class‐switched IgG+ MBCs was significantly lower in the early post‐alloHCT recipients than in healthy controls (Figure 3b). IgM+ S‐specific B cells displayed a similar pattern, with significantly lower frequencies in the early alloHCT recipient group than in healthy controls (Figure 3c). The fraction of RBD‐binding out of total S‐specific IgG+ B cells was similar between alloHCT groups and controls (Figure 3d). Single S/RBD‐specific IgG+ B cells were index‐sorted, and the immunoglobulin heavy chains sequenced to estimate clonality and degree of SHM. SHM of S‐specific BCR sequences was highly variable between individuals (Supplementary figure 6A), and overall similar when comparing pooled sequences from the different study groups, albeit with a slightly higher median level in the < 12mo alloHCT group at the clonotype level (Figure 3e). Clonotype analysis of sorted S+ single cells showed a highly polyclonal response, with occasional expanded clones consisting of > 10 highly similar sequences (Figure 3f). S‐specific sequences frequently used IGHV genes IGHV3‐30, IGHV3‐30‐3, IGHV1‐69 and IGHV3‐15 (Supplementary figure 6B and C), in line with previous data.^26^, ^27^, ^28^, ^29^, ^30^ In summary, < 12 months post alloHCT recipients developed lower levels of S‐ and RBD‐specific peripheral B‐cell memory. However, S‐specific BCR sequences from class‐switched B cells indicated that BCR sequences from HCT recipients were polyclonal and accumulated SHM to at least a similar level as in healthy controls. Taken together, these data indicate a quantitative, but not necessarily qualitative, restriction of the memory B‐cell vaccine response in alloHCT recipients vaccinated < 12mo post transplant.

### Early and late HCT recipients elicit antigen‐specific B‐cell repertoires with similar diversity as healthy controls in response to mRNA vaccination

To improve coverage of the circulating S‐specific B cell pools in alloHCT recipients and better estimate clonal composition and diversity, we queried our bulk IgG and IgM libraries for alloHCT recipients (*n* = 15, excluding one COVID‐19‐experienced individual) and controls (*n* = 4) for sequences highly similar to antigen‐specific clonotypes identified through the single‐cell sequencing described above (Figure 3g). Criteria for identification as antigen‐specific were identical HV‐HJ gene pairing, identical HCDR3 length and 80% amino acid sequence identity in the HCDR3 to at least one sequence in the single‐cell reference dataset. S‐specific clonotype size (Figure 3h) and IGHV SHM (Figure 3i) were similar between alloHCT recipients (*n* = 15) and healthy controls (*n* = 4), irrespective of the 12‐month cut‐off in time elapsed from transplantation and first vaccination. One S‐specific clonotype comprising >5% (approx. 7%) of total repertoire was identified in an alloHCT recipient (Supplementary figure 7A and G). Diversity analysis of antigen‐specific B‐cell clones using the Chao1 index indicated that alloHCT recipients were able to develop BCR repertoires with similar diversity as healthy controls in response to vaccination (Figure 3j).

### Proportion of transitional B cells in alloHCT recipients correlates negatively to vaccine response

To identify potential factors underlying the variable vaccine responses in alloHCT recipients, we extended our study by performing detailed phenotyping of B cells in a subset of participants at study start (Day 0). Participants with a range of vaccine responses in the < 12 and > 12 months post alloHCT groups were analysed (*n* = 35 HCT, 2 CAR‐T, 7 HC).

**Figure 4:**
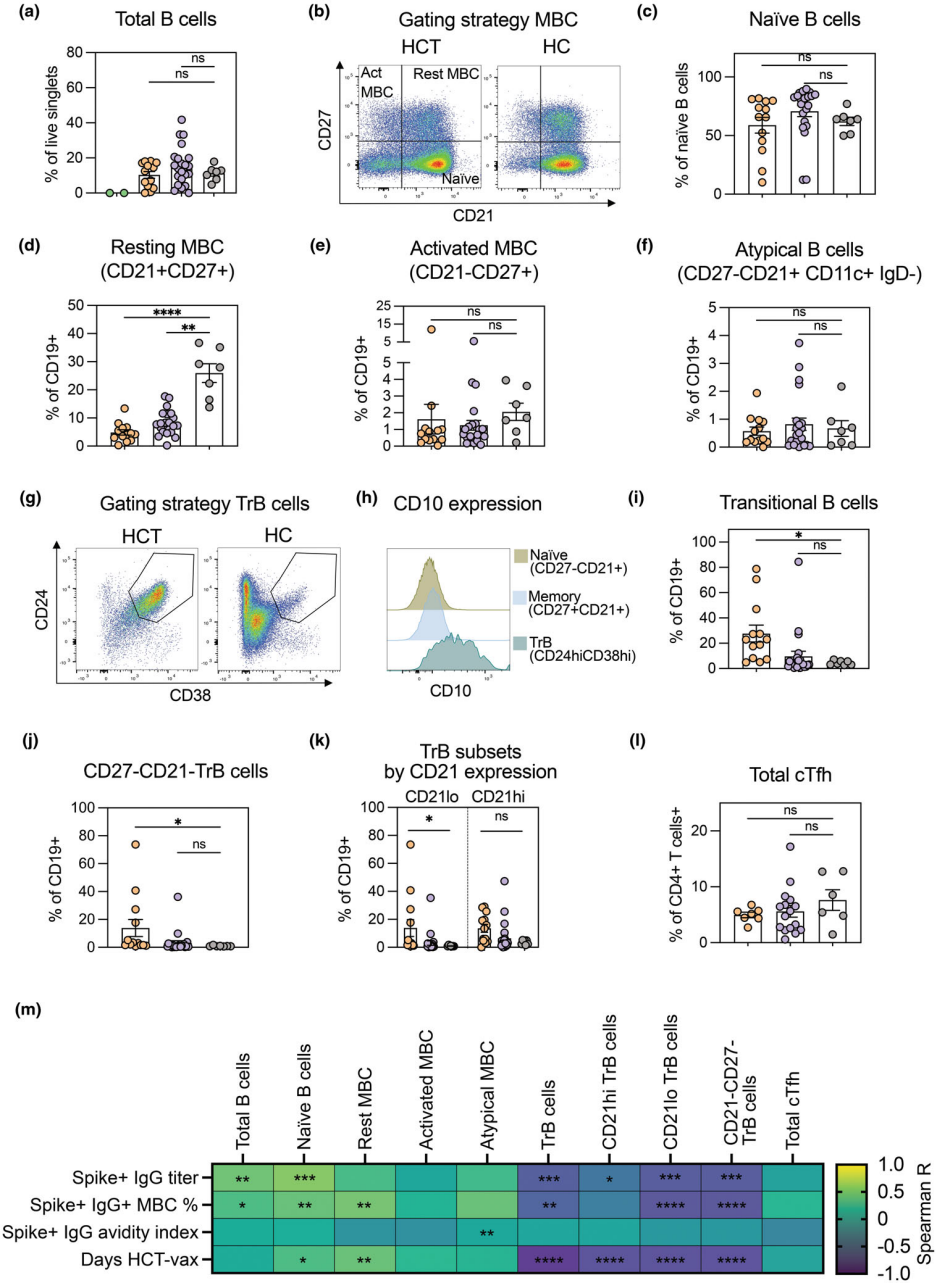
**(a)** Total CD19^+^ B cells shown as percentage of total live single cells. **(b)** Gating strategy for detection of naïve and memory B cell subsets. Rest MBC = Resting memory B cells. Act MBC = Activated memory B cells. **(c–f)** Quantitation of naïve B cells **(c)**, resting memory B cells **(d)**, activated memory B cells **(e)**, and atypical B cells **(f)**, shown as percentages of total CD19^+^ B cells. **(g)** Gating strategy for identification of CD24hi CD38hi transitional B cells (TrB). **(h)** Example of comparative CD10 staining for naïve, resting memory, and transitional B cell subsets. *X* axis shows CD10^−^BUV395 fluorescence intensity. *Y* axis shows event count, scaled as percentage of maximum count (modal axis). **(i)** Quantitation of TrB cells, shown as proportion of total CD19^+^ B cells. **(j, k)** Comparison of CD21hi and CD21lo TrB subsets, expressed as percentages of total CD19^+^ B cells. **(l)** Total cTfh cells in peripheral blood, expressed as percentage of total CD4^+^ T cells. **(m)** Non‐parametric Spearman correlation between Day 0 proportions of TrB cells (% of total CD19^+^ B cells) and day 35 Spike‐specific IgG in serum/plasma (ED50). All groupwise statistical comparisons by Kruskal‐Wallis test with Dunn's *post hoc* test comparing patient groups to healthy controls, unless otherwise specified. *N* = 2 CAR‐T, 35 alloHCT, 7 HC. Where present, error bars indicate mean ± SEM. *P‐value < 0.05, **P‐value < 0.01, ***P‐value < 0.001, ****P‐value < 0.0001.

Relative proportions of total CD19^+^ B cells were not significantly different between the alloHCT groups and healthy controls, but were clearly reduced in the CAR‐T cell recipients (significance not tested due to low *n*), as expected (Figure 4a). Both subgroups of alloHCT recipients had similar proportions of B cells with a CD27^−^, CD21^+^ resting naïve phenotype compared to the healthy controls (Figure 4c). The healthy controls had higher proportions of resting CD27^+^, CD21^+^ memory B cells compared to healthy controls (Figure 4d). We observed elevated percentages of activated memory (CD27^+^CD21^+^) (Figure 4e) and atypical (CD27^−^CD21^+^CD11c^+^IgD^−^) B cells (Figure 4f) in a few alloHCT recipients, but proportions did not differ significantly between alloHCT and healthy controls at the group level. There was no clear influence of cGvHD on the frequencies of B cell subpopulations at Day 0, except for the observation that cGvHD+ alloHCT recipients <12mo from transplant differed more from healthy controls in proportions of CD21^+^ CD27^+^ resting memory B cells than cGvHD‐ (Figure S8C). A trend was seen towards higher proportion of TrB in cGvHD^+^ < 12mo alloHCT group than that of controls (Supplementary figure 8F), but the difference was not significant. Comparison of alloHCT recipients with and without ongoing immunosuppression showed that alloHCT recipients > 12mo without immunosuppression had slightly higher proportions of naïve B cells (Supplementary figure 9B) than that of controls.

As transitional B cells (TrB cells) have been reported to be elevated in alloHCT recipients early after transplantation,^11^, ^16^, ^31^, ^32^, ^33^ we quantified the proportions of TrB cells, identified as CD24hi CD38hi CD19^+^ cells (Figure 4g, Supplementary figure 15). During assay establishment, we confirmed that the CD24hi CD38hi TrB population expressed higher levels of CD10 (MFI) than that of populations identified as resting naïve B cells and resting memory B cells (Figure 4h). We found that proportions of TrB cells were significantly elevated in alloHCT recipients < 12 months after transplantation compared with healthy controls, although there was a high degree of variability within both the < 12 and > 12 months alloHCT groups (Figure 4i). Within the TrB population, group differences were most pronounced in the CD27^−^CD21^−^ (Figure 4j) or CD21lo (Figure 4k) compartments. CD21lo TrB cells have been described as more functionally immature than CD21hi.^33^

Where cell numbers allowed, we additionally assessed the proportions and phenotypes of T cells, including circulating T‐follicular helper cells (cTFH). AlloHCT recipients showed similar levels of total T cells as healthy controls (Supplementary figure 10A). The CD4/CD8 ratio was decreased in alloHCT recipients (Supplementary figure 10B and C), though the proportion of cTFH cells out of CD4^+^ was similar to that of healthy controls (Figure 4l).

To understand the impact of the peripheral immune cell pool composition on the response to vaccination, we correlated proportions of different B‐cell subpopulations at the study start to the level of S‐ and RBD‐specific IgG attained after two vaccine doses (Day 35). There was a moderate negative correlation between fractions of TrB cells and S‐specific antibodies IgG (Day 35) (Figure 4m). TrB was also negatively correlated to time elapsed post‐alloHCT (Figure 4m). To better understand the relative contributions of TrB and clinical parameters, a multivariable linear regression analysis including time since transplantation, immunosuppression status and TrB frequency was performed. The TrB emerged as the strongest independent predictor of vaccine antibody titres (Supplementary figure 11), indicating that baseline transitional B‐cell frequency is a robust determinant of post‐vaccination antibody responses in this cohort.

### AlloHCT recipients show intact B‐cell differentiation potential

**Figure 5:**
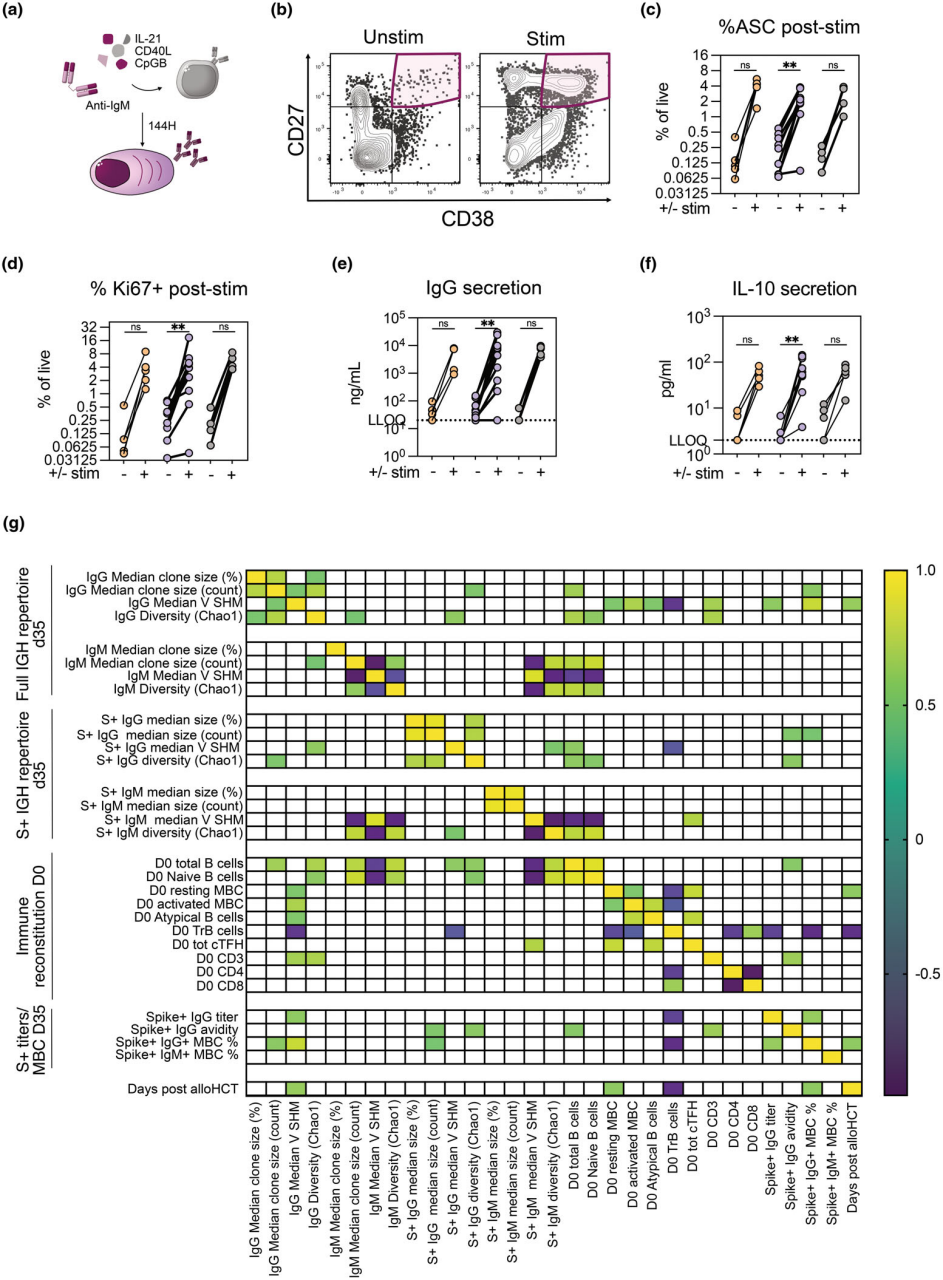
**(a)** Experimental schematic for *in vitro* differentiation of PBMC to ASCs by *ex vivo* stimulation. **(b)** Gating strategy of identification of antibody‐secreting cells (ASCs) with and without extrinsic stimulation. **(c)** %ASCs in 6‐day cultures of PBMC in the presence (stim) or absence (unstim) of mouse‐anti‐human IgM Fab(2) fragments, CpGB, soluble CD40L and IL‐21. **(d)** %Ki‐67+ B cells in 6‐day cultures of PBMC in the presence (stim) or absence (unstim) of mouse‐anti‐human IgM Fab(2) fragments, CpGB, soluble CD40L and IL‐21. **(e)** Concentration of IgG in 6‐day stimulated culture supernatants, quantified by ELISA. Unstimulated‐stimulated pairwise comparison by Wilcoxon signed rank test. **(f)** Concentration of IL‐10 in 144 h stimulated culture supernatants, quantified by ELISA. *N* = 21 (15 alloHCT, 5 HC) for 3E‐F, 3I‐J. *N* = 19 (14 alloHCT, 4 HC) for 3C‐D. One alloHCT and one HC excluded because of technical problems with flow cytometry analysis. **(g)** Non‐parametric Spearman correlations of Day 0 B cell phenotypes to vaccine immunogenicity and immunoglobulin repertoire features measured at Day 35 (post prime and boost mRNA vaccination). *N* = 15 (healthy controls excluded from correlation analysis).

We performed functional *in vitro* cultures of PBMCs from alloHCT recipients to measure whether their B cells had intact capacity to respond to stimulation, differentiate to antibody‐secreting cells and produce soluble antibody. PBMCs from a subset of < 12 months and > 12 months alloHCT recipients (*n* = 15), as well as from healthy controls (*n* = 5) were cultured with or without addition of anti‐human IgM Fab^2^ fragments, IL‐21, CD40L and CpGB for 144 h (6 days) (Figure 5a). Cultured cells were analysed for expression of CD27 and CD38, indicative of differentiation to the antibody‐secreting cell (ASC) state (Figure 5b). Selection of samples for *ex vivo* stimulation was based on the availability of PBMCs as well as B‐cell levels (participants expected to have near‐total B‐cell aplasia were not selected). Polyclonal activation induced similar levels of ASC differentiation (CD19^+^CD38hiCD27hi) (Figure 5c, Supplementary figure 12C), Ki‐67 expression in CD19^+^ cells (Figure 5d, Supplementary figure 12D) as well as IgG (Figure 5e, Supplementary figure 12E) and IL‐10 (Figure 5f, Supplementary figure 12F) production in the assessed alloHCT recipients compared to healthy controls. The fold change in IgG concentration was slightly lower in some alloHCT recipients than in healthy controls (Supplementary figure 12E); however, substantial antibody production was induced in all tested samples except one. There was a moderate correlation between the proportion of CD21hi TrB cells (as observed in phenotypic analysis in Figure 4i) and the fold change of ASCs after polyclonal activation (Supplementary figure 12G). We also observed a correlation between frequency of resting memory B cells and induction of IgG in stimulated culture supernatants (fold change compared to unstimulated controls) (Supplementary figure 12G). In summary, PBMC from both the < 12 and > 12 months post alloHCT recipients tested in this assay showed similar capacity to differentiate toward ASC phenotype and produce antibodies as cells from healthy controls. These results suggest that B cells in the tested alloHCT recipients were functional when provided with sufficient stimuli.

### Proportion of TrB cells negatively correlates with IGHV SHM but not to BCR diversity

To assess whether there is a link between B‐cell phenotype, BCR repertoire composition and vaccine responses, we performed correlation analysis using datasets from Day 0 and Day 35. Diversity (Chao1) of the Day 35 S+ IgG pool was positively correlated with the Day 35 antibody avidity, as well as the pre‐vaccination proportions of total and naïve B cells (Figure 5g). Similar to IgG, total IgM diversity was positively correlated with the proportions of total and naïve B cells. Both total and S‐specific IgG IGHV SHM negatively correlated with baseline proportions of transitional B cells, while total IgG IGHV SHM positively correlated with baseline proportions of activated and resting memory B cells (Figure 5g). There was no significant correlation between IgG diversity and TrB cells. Taken together, these data support the conclusion that while mRNA vaccination of alloHCT recipients can in many cases elicit a highly diverse antigen‐specific B‐cell repertoire with IGHV mutational load similar to that of a control population, the presence of immature B‐cell populations may limit SHM accumulation.

## Discussion

A prospective European study demonstrated that 236 patients with SARS‐CoV‐2 infections after alloHCT had a significant mortality risk with survival rates as low as 78% 6 weeks after diagnosis of COVID‐19.^34^ Thus, alloHCT recipients have been a prioritised group for vaccination as a protective intervention.^35^ We and others have demonstrated that SARS‐CoV‐2 mRNA vaccines are safe and immunogenic in a large fraction of alloHCT recipients.^3^, ^8^, ^36^, ^37^, ^38^, ^39^ With vaccines and new treatments arriving and new variants of SARS‐CoV‐2 surfacing, overall survival improved markedly over time.^40^ Improved survival has also been shown for CAR‐T recipients.^41^ However, mechanistic data on the functional characteristics and diversity of vaccine‐elicited immune responses in patients after alloHCT are still lacking. In this study, we performed a detailed characterisation of the antibody and B‐cell response to SARS‐CoV‐2 mRNA vaccination in alloHCT recipients, with the aim of understanding whether vaccine‐elicited immune responses in this patient population differ in functional characteristics compared with a control population. Our results reaffirm previous reports, showing that mRNA vaccination induced antibody responses to SARS‐CoV‐2 in a majority of alloHCT recipients, and that the magnitude of the antibody response correlated with the time elapsed from alloHCT. Through detailed immunological analyses, we observed that alloHCT recipients who developed detectable antibody responses to vaccination showed functional responses in terms of neutralising capacity. Antibody responses were overall similar in avidity and cross‐reactivity compared with healthy controls. This indicates that antibody responses elicited in alloHCT recipients can acquire functional characteristics similar to those observed in a healthy control population. Indeed, vaccination has also been shown to be successful in reducing mortality; for example, a study including almost 1000 alloHCT patients reported that fully vaccinated patients who contracted COVID‐19 had a mortality of only 1%.^40^

Few studies have characterised the B‐cell pool following alloHCT at the level of BCR clonal composition. Persistent reduction in diversity in class‐switched VH1 BCRs in the bone marrow compared with pre‐transplant samples has been demonstrated, but other IGHV gene families were not assessed.^42^ In elderly populations, limited BCR diversity has been associated with increased frailty.^43^ In our study, we found that the IGHV and IGHJ gene usage in the overall IgG and IgM repertoires were similar between alloHCT recipients and healthy controls, with similar median clonal size and receptor diversity (the latter quantified by Chao1 and ACE indexes). These data indicate that alloHCT recipients had a highly polyclonal B‐cell pool, that was not restricted in terms of IGHV and IGHJ usage. We noted that IgG repertoires of some alloHCT recipients contained clones that constituted a significant portion of the overall repertoire, a pattern not observed in the IgG repertoires of the healthy controls or in the IgM repertoires of either group. The presence of expanded clones among class‐switched B cells aligns with prior data describing a more oligoclonal B‐cell repertoire early after alloHCT in memory B cells, but not naïve B cells.^44^ The specificities of these expanded clones require further investigation; efforts on our part to query our IgG repertoires with published sequences of known pathogen specificity yielded no matches, though this may be simply because of the vast inter‐individual variability of immunoglobulin sequences. In the light of evidence that B cells contribute to both GvHD and GvL, it is possible that these expanded BCR clones are specific to alloantigens; however, our dataset did not show a clear association of expanded B‐cell clones and the presence of chronic GvHD. We also found that median V‐SHM of the IgG repertoire was reduced in early alloHCT recipients compared with healthy controls and that the degree of IgG SHM was related to the time elapsed from alloHCT to study start. This likely reflects a time‐dependent increase of overall mutational load as new germinal center responses are induced by antigen exposures that occur post‐alloHCT (e.g. infections, immunisations). Previous studies have shown the influence of chronic GvHD on delaying the reconstitution of the B‐cell repertoire.^16^, ^45^ We did not observe a clear effect of cGvHD on median repertoire V‐SHM, though our small sample size may limit the sensitivity of the comparison.

Our quantitative analysis of the B‐cell vaccine response in alloHCT recipients revealed that S‐specific MBCs were detectable in a large proportion of both early and late alloHCT recipients, and notably also for one CAR‐T recipient. However, as observed for S‐specific IgG, the memory response was highly heterogeneous, with non‐responders present in both the early and late alloHCT groups. Preferential usage of certain VH genes and skewed SHM have been documented for antigen‐specific BCR repertoires in infancy^46^ and old age,^47^, ^48^ indicating that immunological fitness has bearing on the vaccine‐specific B‐cell repertoire. In our cohort, individuals who developed a detectable S‐specific MBC response showed a highly polyclonal response with overall similar VH‐gene usage and SHM in alloHCT recipients compared with healthy controls. For the subset of study participants where bulk IgG repertoire sequencing was performed, we queried bulk IgG sequencing data using our single‐cell S+ BCR dataset to achieve higher repertoire coverage and better estimate S‐specific clonal distribution and BCR diversity. In this subset of alloHCT recipients, we again observed an S‐specific BCR pool with similar SHM and clonal expansion as seen in healthy controls. AlloHCT recipients also showed similar diversity of the S‐specific IgG repertoire compared with the control group. Taken together, these data indicate that alloHCT recipients who develop detectable MBC responses to mRNA vaccination are able to generate antigen‐specific memory B cells with qualitative characteristics similar to healthy controls. While lower frequencies of S+ MBC seen in some alloHCT recipients may negatively impact recall of vaccine‐induced memory, presence of high‐affinity B cells, and T cell help, may mitigate this effect.

While we observed a clear relationship between time elapsed post‐alloHCT and the magnitude of the measured antibody and MBC responses, we noted that this relationship was heterogeneous, with high responders present in the < 12mo group and alloHCT recipients who were non/low‐responders despite > 2 years elapsed post‐transplant. We sought to identify deficiencies in immune reconstitution that affect vaccine responsiveness. B‐cell aplasia has been previously identified as a predictor of poor seroconversion to mRNA vaccination in alloHCT recipients.^7^ Our phenotypic analysis revealed that early alloHCT recipients displayed elevated frequencies of immature TrB cells in peripheral blood. The proportion of TrB cells, especially the CD21^−^ low subset, negatively correlated with the magnitude of the S‐specific antibody and MBC response. The proportion of TrB cells at Day 0 of vaccination correlated negatively to levels of SHM in the total and spike‐specific IgG repertoires observed post‐vaccination, but did not have any apparent correlation to overall IgG or IgM receptor diversity. These results indicate that deficient vaccine responses in alloHCT recipients may arise from functional limitations rather than restricted clonal diversity. Previous studies have documented regulatory B cell subsets within the CD24hiCD38hi population here identified as TrB cells^49^ and cGvHD has been associated with lower levels of regulatory B cells.^50^ Though our study does not delve into this aspect, it could be speculated that regulatory functions within the TrB population may exert suppressive effects on the vaccine response. However, we did not observe a correlation between TrB proportions and IL‐10 production in our *ex vivo* stimulation assays. Factors that correlated positively with the vaccine response included the fraction of total B cells out of live cells, as well as the proportions of naïve B cells and resting MBC out of the total CD19^+^ pool. In summary, the phenotypic reconstitution of the B‐cell pool appears to influence the magnitude of the vaccine response in alloHCT recipients, where elevated levels of immature TrBs may limit the capacity to develop robust antigen‐specific humoral immunity.

In conclusion, our findings highlight the intricacies of B‐cell reconstitution following alloHCT and contribute to unravelling the considerable variability in vaccination responses within this patient population. Our study demonstrates the impact of B cell reconstitution on immune responses to vaccination, and contributes one of few characterisations of the post‐alloHCT reconstituting BCR sequence repertoire. Limitations include the sample size (of note, data on CAR‐T recipients should be interpreted with particular caution given low *n*), limited availability of specific data types across the cohort, and the clinical heterogeneity of the alloHCT recipient group, complicating conclusive analysis of cause‐and‐effect relationships. There is also an inherent bias when isolating antigen‐specific B cells from a heterogenous cohort, as only individuals with detectable cells can be analysed and high responders permit collection of a larger number of sequences and the generation of more robust data. Similarly, our serum epitope binning and avidity analyses are not possible to perform on antibody responses falling below limit of detection. While our study provides evidence supporting proportions of TrB as a potential predictive marker of vaccine responses in alloHCT recipients, independent validation is needed, including investigation of responses to other vaccines. Further studies are also needed to determine whether a causal relationship between TrB and vaccine responsiveness exists post‐alloHCT.

## Patients and methods

### Study approval

The study participants described here were part of a previously published prospective clinical trial, COVAXID.^36^ This trial was registered at EudraCT (no. 2021‐000175‐37) and clinicaltrials.gov (NCT04780659). The Swedish Medical Product Agency (ID 5.1‐2021‐5881) and the Swedish Ethical Review Authority (ID 2021‐00451) approved the study. The original clinical trial protocol included two vaccine doses and immunogenicity measurements until 6 months after the second dose and was subsequently extended with permission from the Swedish Ethical Review Board and the Swedish Medical Products Agency (no. 2021‐06046‐02 and no. 5.1‐2021‐92 151, respectively). The primary endpoint was seroconversion after two vaccine doses at Day +35 from first vaccination. Immune reconstitution was a secondary endpoint. Study participants who did not receive their second immunisation were excluded from analysis. Study participants who had pre‐existing immunity at baseline, a positive PCR at baseline, or PCR‐verified COVID‐19 infection between Day 0 and Day 35 were considered COVID‐19+. These participants are reported separately in Figure 1 and are excluded from further analysis to avoid misconstruing the assessment of vaccine immunogenicity. The exception to this is one COVID‐19+ participant who was included in the immunoglobulin repertoire sequencing analysis and is reported in Figure 2 (which pertains to the overall IGH repertoire), but is not included in the Spike+ B‐cell repertoire analysis in Figure 3.

### Sex as a biological variable

Both male and female alloHCT recipients were included in the study. We included both male and female participants in all sub‐selections made for specific analyses. Because selection was also based on cGvHD^+^ vs. cGvHD^−^ and sample availability, we were not always able to maintain a 50‐50 ratio of male–female participants, for example in the case of BCR repertoire sequencing experiments. However, our assessment is that we have a relatively well‐balanced study population in terms of biological sex. For this study population, we do not expect the sex of study participants to have significant bearing on alloHCT engraftment or cGvHD. Prior data have demonstrated sex‐dependent differences in both innate and immune functions, as reviewed by Klein *et al*.^51^ and Dunn *et al*.^52^ In the context of vaccination, female sex has been associated with higher antibody responses to influenza vaccination^53^ and mouse models have shown greater diversity of germinal centre B cells after influenza immunisation.^54^ Conversely, male sex has been associated with higher disease severity of COVID‐19.^55^ However, for mRNA SARS‐CoV‐2 vaccination, early efficacy studies of the BNT162b2 (Pfizer/BioNTech)^56^ and mRNA‐1273 (Moderna Therapeutics)^57^ showed similar vaccine efficacy for male and female participants. The previously reported quantitative analysis of Spike‐specific antibody responses in the Phase IV clinical trial by Bergman *et al*.,^3^ which includes the study population reported here, also did not demonstrate significant impact of biological sex on antibody titres at Day 35.

### Sample processing

Samples from Day 0 and Day 35 were collected according to the study protocol described in Bergman *et al*.^3^ Briefly, samples were collected into sodium heparin Vacutainer tubes (BD Biosciences). For plasma separation, a small aliquot of whole blood was centrifuged at 500 g for 10 min, and the plasma fraction was collected and stored at −20°C until use. Whole blood was diluted 1:1 with phosphate‐buffered saline (PBS) layered onto a Lymphoprep (Stemcell) or Ficoll‐Paque (Cytiva) gradient in SepMate tubes (Stemcell) according to manufacturer instructions. A 10‐min centrifugation at 1200 g was used for separation of PBMC from diluted whole blood. The PBMC‐containing fraction was transferred to a clean tube and washed twice with phosphate‐buffered saline (PBS) containing 2% heat‐inactivated fetal bovine serum (FBS). PBMCs were resuspended in PBS and counted using trypan blue and an automated cell counter. PBMC were cryopreserved in FBS containing 10% dimethylsulfoxide (DMSO) and stored frozen at −180°C until use.

### Assessment of SARS‐CoV‐2 specific IgG responses in plasma and serum

Prior to serological assessment, plasma/serum samples were incubated at 56°C for 30 min. ELISA analysis for quantitation of S‐ and RBD‐specific antibodies was carried out at previously described,^58^, ^59^ with modifications. Half‐area ELISA plates (Greiner Bio‐One or Corning) were coated at 4°C overnight with 50 ng well^−1^ of either SARS‐CoV‐2 HexaPro Spike proteins or soluble RBD (kind gifts of Neil King, University of Washington), at 1 μg mL^−1^ in PBS. ELISA plates were washed 3× using PBS containing 0.05% Tween‐20 (PBS‐T); all subsequent washing steps were performed identically unless otherwise specified. Plates were blocked for 1 h at room temperature (RT) using 5% (w/v) milk powder in PBS. Plasma or serum samples were diluted 5‐fold in PBS with 5% milk. 50 μL serially diluted samples was added to ELISA plates and incubated for 2 h at RT, followed by a wash step. For assessment of antibody avidity by chaotropic ELISA, parallel plates were treated with either PBS or 1.5 M NaSCN, 50 μL well^−1^, for 10 min followed by washing. Subsequently, plates were incubated 1 h at RT with 50 mL well^−1^ peroxidase‐conjugated anti‐human‐IgG or anti‐IgA secondary antibody (Jackson Immuno) diluted 1:5000 (IgG) or 1:2000 (IgA) diluted in PBS + 5% milk. Plates were then washed. For detection, 50 mL well^−1^ 1‐step Ultra TMB substrate (Thermo Fisher) was added and incubated for 5 min at RT. Peroxidase activity was stopped using 50 mL well^−1^ 1 m H_2_SO_4_. Plates were read at 450 nm with 570 nm background correction using a VarioSkan Lux Multimode reader (Thermo Fisher). For IgG binding titres and avidity assessments, plates were read at 450 nm with 550 nm background correction using an EnSpire Multilabel Reader (PerkinElmer). Optical density (OD) at 570/550 nm was subtracted from the OD at 450 nm and the resultant OD was used in all subsequent calculations. A four‐point logarithmic (4PL) non‐linear regression curve fit was performed using Graphpad Prism version 9.1 (San Diego, California). Titres giving half‐max OD (ED50) was used as readout for plasma. For assessment of SARS‐CoV‐2 Spike‐specific avidity, the avidity index was calculated as the percentage of binding remaining in 1.5 m NaSCN treated plates as compared with untreated plates: ED50(NaSCN)/ED50(PBS) × 100%. For assessment of binding to SARS‐CoV‐2 variants of concern, the ratio of ED50 (variant spike) to ED50 (ancestral spike) was used as readout.

### Assessment of SARS‐CoV‐2 specific IgG epitope targeting by monoclonal antibody competition ELISA

For mAb competition ELISA, monoclonal SARS‐CoV‐2 Spike‐binding antibodies were biotinylated using the EZ‐Link Sulfo‐NHS‐LC‐biotin kit (Thermo Fisher) according to manufacturer instructions. ELISA was performed as above with the following changes: Plates were coated with recombinant Spike protein at 1 μg mL^−1^, or 2 μg mL^−1^ for CR3022 competition. Plasma/serum was serially diluted threefold with a starting dilution 1:5. Samples were incubated for 30 min at RT, after which a fixed concentration of biotinylated monoclonal diluted in 5% milk buffer was added and incubated a further 90 min at RT. Plates were then washed as described for binding ELISA, and incubated with NeutrAvidin‐HRP (Thermo Fisher) at 1:5000 in PBS for 60 min at RT. Plates were washed, developed using TMB, and read as described above. A standard curve of monoclonal antibody in competition with its biotinylated counterpart was run in parallel with each sample batch. A 4‐point logarithmic non‐linear regression curve fit was performed using Graphpad Prism 10 for Mac (Boston, Massachusetts, USA) for each sample, and the ED50 used as read‐out. Curve fits of poor quality (visual assessment) or with competition too low for accurate quantitation were excluded from analysis. ED50 < LLOQ (1:5) were adjusted to LLOQ for statistical comparison.

### Neutralisation assays

Live virus neutralisation assay was performed as previously described^60^ with minor modifications. Briefly, SARS‐CoV‐2 wild‐type strain (SARS‐CoV‐2/01/human/2020/SWE) was grown on Vero E6 cells. For virus neutralisation assay, samples were serially diluted fivefold in serum‐free Dulbecco's Modified Eagles Medium (DMEM) supplemented with 0.2% penicillin/streptomycin. Diluted serum samples were incubated with 1000 PFU well^−1^ SARS‐CoV‐2 virus for 30 min at 37°C. Following pre‐incubation, serum‐virus mix was added to Vero E6 cells, seeded in Greiner CELLSTAR® 96‐well plates (Greiner Bio‐One, Austria) at a density of 10^4^ well^−1^, 1 day prior to the experiment. Cells were infected for 8 h, then fixed in 4% formaldehyde for 40 min. Plates were washed with PBS, and cells permeabilised for 10 min at RT using 0.5% Triton‐X and 20 mm glycine in PBS. Plates were blocked for 30 min at RT using 2% bovine serum albumin (BSA) in PBS followed by staining of infected cells using a rabbit‐anti‐SARS‐CoV‐2 nucleocapsid antibody (Sino Biological) for 1 h at RT, followed by a goat‐anti‐rabbit IgG (H + L) AF‐488 secondary antibody for 30 min at RT. Plates were counter‐stained with DAPI (0.1 mg mL^−1^) for 10 min. Fluorescent signal was measured using a TROPHOS plate RUNNER HD instrument (TROPHOS SA, Marseille, France). The half‐maximal inhibitory dilution reciprocal (ID50) was determined by 4‐parameter nonlinear regression performed in Graphpad Prism 9.0 (Boston, Massachusetts, USA).

### B‐ and T‐cell phenotype analysis

Frozen PBMC were thawed in a water bath at 37°C, resuspended in R10 medium, washed twice (1500 RPM × 5 min), rested for 1–3 h in an incubator at 37°C with 5% CO_2_ and counted.

1–3 × 10^6^ PBMC were collected for B‐ and T cell phenotyping. PBMC were stained using one of two panels of fluorescently labelled antibodies. For B‐cell phenotyping: CD19^−^ECD (J3‐119, Beckman Coulter), CD10^−^BUV395 (MEM‐78, BD Biosciences), CD11c^−^AF700 (3.9, BioLegend), IgM‐PerCP‐Cy5.5 (G20‐127, BD BioSciences), CD38^−^APC‐Cy7 (HIT2, BioLegend), CD21^−^BV711 (B‐ly4, BD Biosciences), CD123^−^BV510 (6H6, BioLegend), CD3^−^BV510 (SP34‐2, BD Biosciences), CD24^−^BV650 (ML5, BD Biosciences), CD27^−^PE‐Cy7 (M‐T271, BioLegend), IgG‐BV786 (G18‐145, BD Biosciences), CD16^−^BV510 (3G8, BD Biosciences), CD56^−^BV510 (B159, BD Biosciences), CD20^−^BV605 (2H7, BioLegend), CD14^−^BV510 (M5E2, BioLegend), IgD‐BV488 (Polyclonal, Southern Biotech) and 7AAD live/dead viability stain (Invitrogen). Surface staining was performed at 4°C for 20 min. Cells were washed using PBS‐FCS and permeabilised using the BD Transcription Factor Buffer kit (BD Biosciences). Cells were resuspended in 200 μL of kit Fix per Perm buffer and incubated for 20 min at RT. Cells were thereafter washed with the kit perm/wash buffer and stained intracellularly using a panel of intracellular antibodies Ki‐67‐PE (B56, BD Biosciences), IRF4‐Pacific Blue (IRF4.3E4, BioLegend), BLIMP‐1‐AF647 (6D3, BD Biosciences) diluted in perm/wash. Cells were washed using perm/wash buffer, resuspended in PBS containing 1% FA, and acquired using a BD LSRFortessa flow cytometer. For T cell and monocyte phenotyping, surface staining was performed using the following markers: CCR6‐BV786 (11A9, BD Biosciences), CXCR3 BUV395 (1C6, BD Biosciences), CXCR5 E‐Fluor610 (MU5UBEE, Invitrogen), CD38 AF488 (HIT2, BioLegend), CD3 APC‐Cy7 (SP34‐2, BD Biosciences), CD16 AF700 (3G8, BD Biosciences), CCR7 BV421 (G043H7, BioLegend), CD20 BV510 (2H7, BioLegend), CD19 BV510 (HIB19, BD Biosciences), CD56 BV510 (B159 BD Biosciences), HLA‐DR BV510 (L243 BioLegend), CD4 PE‐Cy5 (SK3 BD Biosciences), CD8a BV711 (RPAT‐T8, BioLegend), ICOS PerCP‐Cy5.5 (C398.4A, BioLegend), CD14 AF677 (M5E2, BioLegend), CD45RA BV650 (5H9, BD Biosciences), and intracellular staining using FoxP3 PE (206D, BioLegend). Data were analysed using Flowjo version 10. Gating strategies used for the identification of B‐cell, T‐cell and monocyte populations shown in Supplementary figure 15.

### *Ex vivo* polyclonal B cell activation assay

Frozen PBMC were thawed as described for phenotypic analysis above. After aliquoting of PBMC for phenotyping, samples with sufficient remaining PBMC were resuspended in R10 medium at 10 × 10^6^ cells mL^−1^. Cells were then cultured for 144 h in the presence or absence of polyclonal activators as previously described,^61^ with modifications. Stimulation was performed in R10 medium containing 0.5 μg mL^−1^ anti‐IgM Fab2 fragments (Southern BioTech), 1 μg mL^−1^ soluble CD40L (Stemcell), 50 ng mL^−1^ IL‐21 (Stemcell) and 2.5 μg mL^−1^ CpGB class B ODN (Invivogen). Briefly, 100 μL of cell suspension containing 1 × 10^6^ cells were plated onto 48‐well treated tissue culture plates (Corning‐Falcon). One to five replicate wells were plated per sample and condition depending on sample availability. Four hundred microliters of R10 containing polyclonal activators was added to stimulated wells. Four hundred microliters R10 without activators was added to control wells. Cells were cultured for 144 h (6 days), after which replicate wells were pooled in FACS tubes and centrifuged (450 *g* × 5 min). Supernatants were collected and stored frozen at −20°C for later analysis by ELISA. Cultured PBMC were washed with PBS containing 2% FCS and stained for surface and intracellular markers using the same antibody panel described above for phenotyping. Gating strategy used for the identification of CD27^+^CD38^+^ ASCs and Ki67^+^ B cells is shown in Supplementary figure 16.

### IgG and IL‐10 quantitation in culture supernatants

For detection of total IgG, an in‐house sandwich ELISA set‐up was used. Briefly, half‐well ELISA plates (Greiner BioOne) were coated with 2 μg mL^−1^ anti‐human IgG antibody (clone MT145) overnight at 4°C. Plates were washed 3× with PBS‐T and blocked for 1 h at RT with 5% milk powder in PBS. Blocking buffer was removed and supernatant samples diluted in 5% milk‐PBS were added to plates. An 11‐point standard curve of human IgG (Sigma‐Aldrich) was run in each plate alongside samples. Samples were incubated for 2 h at RT. Plates were washed 3× with PBS‐T and 50 μL horseradish peroxidase conjugated goat‐anti‐human IgG (Jackson Immuno) diluted 1:7500 in 5% milk‐PBS was added to wells and incubated 1 h at RT. Plates were washed 3x and developed using 1‐step TMB substrate (Thermo Fisher) for 5 min, after which the reaction was stopped using 1 m H_2_SO_4_. Plates were read at 450 nm with 570 nm background correction using a Varioskan LUX ELISA reader instrument (Thermo Fisher). Background subtracted OD values were used as readout. For detection of IL‐10, the MabTech human IL‐10 (HRP) ELISA Flex kit was used according to manufacturer instructions and using half‐well ELISA plates (Greiner BioOne). Plates were developed and read as described for anti‐IgG ELISA. For both IgG and IL‐10, 5‐point logarithmic standard curve fits and interpolation of sample ODs were performed in Graphpad Prism 10 to calculate concentrations of the respective analytes. Samples below LLOQ were adjusted to LLOQ for fold change calculations.

### Assessment of SARS‐CoV‐2 specific memory B cell responses

Fluorescently conjugated antigen probes were prepared as previously described.^58^, ^59^ Briefly, SARS‐CoV‐2 S2‐P Spike and soluble RBD proteins (kind gifts of Neil King, University of Washington, USA) were biotinylated using Sulfo‐NHS‐biotin (Thermo Fisher) according to manufacturer instructions. Excess biotin was removed using Slide‐A‐Lyzer mini dialysis devices (Thermo Fisher). Prior to staining of PBMCs, fluorescent Spike and RBD tetramers were constructed by stepwise incubation of biotinylated proteins with either Streptavidin‐APC, Streptavidin‐PE or Streptavidin‐BV421 (BioLegend) on ice, for a 4:1 final molar ratio of protein to streptavidin conjugate. Frozen PBMCs were thawed in a 37°C water bath and transferred to RPMI complete medium (HyClone) supplemented with 10% FBS (Gibco), 1% penicillin/streptomycin and 1% L‐glutamine (R10). Cells were washed twice with R10, rested for 1–3 h (37°C, 5% CO_2_), and counted using Trypan blue and an automated cell counter. One to three million PBMCs was transferred to FACS tubes, washed with cold PBS containing 2% FBS and stained with 100 ng each of Spike‐PE tetramer, Spike‐APC tetramer and RBD‐BV421 tetramer for 20 min at 4°C. Cells were then surface stained with anti‐human IgM‐PerCP‐Cy5.5 (G20‐127; BD Biosciences), CD3^−^ BV510 (SP34‐2; BD Biosciences), CD123^−^BV510 (6H6; Biolegend), CD19^−^ECD (J3‐119; Beckman‐Coulter), CD16^−^BV510 (3G8; BD Biosciences), HLA‐DR‐ BV650 (L243; Biolegend), IgG‐BV786 (G18‐145; BD Bioscience), CD20^−^BV605 (2H7; Biolegend), CD14^−^BV510 (M5E2; BioLegend) and IgD‐FITC (Polyclonal; Southern Biotech) for 20 min at 4°C. Samples were washed with cold PBS with 2% FBS, resuspended in R10 media containing 0.25 μg mL^−1^ 7AAD viability dye (Invitrogen) and acquired using a FACSAria III Fusion instrument. Single cell index sorting of Spike‐PE and Spike‐APC double positive IgG+ B cells was conducted simultaneously with data collection. Sorting was performed at the Biomedicum Flow Cytometry Core Facility, which receives funding from the Infrastructure Board at Karolinska Institutet. Cells were sorted dry into 96‐well PCR plates (Bio‐Rad), immediately transferred onto dry ice, and stored at −80°C until downstream use. Data was analysed using Flowjo version 10 (FlowJo Inc.). Representative gatings are shown in Supplementary figure 13A (sorting) and Supplementary figure 13B (quantitative analysis).

### Amplification of VDJ genes by single‐cell PCR and Sanger sequencing

RNA from single Spike‐specific sorted memory B cells was extracted and reverse‐transcribed into cDNA with random hexamers using the Superscript III kit (Invitrogen) following the manufacturer's instructions. Single‐cell nested PCR was then performed using human IgG heavy‐chain VDJ primers previously reported by Doria‐Rose *et al*.,^62^ and using the HotStarTaq Plus Polymerase kit (Qiagen). Raw sequencing data from Sanger sequencing was filtered for quality using the R/Bioconductor package scifer (v 1.0.0) using default settings as described in Arcoverde Cerveira *et al*.^63^ Good quality sequences were aligned using IgDiscover (v 0.15.1)^64^, as implemented in the in‐house pipeline IgXplore,^65^ to the OGRDB AIRR‐C germline gene database.^66^, ^67^ Using 0 iterations and settings for single‐end reads, clonally related sequences were defined using the IgDiscover clonotypes model as those with the same V and J alleles, identical HCDR3 length, minimal 80% aa identity of HCDR3, and at least one junction without a mismatch.

### Bulk BCR library preparation and repertoire sequencing

IgG and IgM libraries for high‐throughput sequencing (HTS) by Illumina MiSeq 2 × 300 bp paired‐end sequencing were based on a 5‐prime multiplex PCR method. The RNA extraction was performed using Rneasy Mini Kit (Qiagen) following the manufacturer's instructions. The cDNA synthesis, multiplex PCR and Index PCR steps were performed following the previously described protocol and primers.^68^ The library pool and final sample preparation for the sequencing were performed using MiSeq Reagent Kit v3, 600 cycles (Illumina) following the protocol described in Vazquez‐Bernat *et al*.^68^

### IgG bulk repertoire sequencing and lineage tracing

Reads processing was performed using the IgDiscover program (v. 0.15.1)^64^, with default settings, except for UMI length of 21 nucleotides and 0 iterations, and using the OGRDB AIRR‐C germline reference database.^66^, ^67^ To integrate the antigen‐specific Sanger sequences with the bulk repertoire, we used the antigen‐specific sequences to query the bulk IgG repertoire sequencing to trace their clonal relatives. To achieve that, we used the IgDiscover *clonoquery* and *clonotypes* modules.^69^ We first identified the clonotypes present among all antigen‐specific sequences identified through Sanger sequencing, using the IgDiscover *clonotypes* function with the same clonotype definition as mentioned previously. Subsequently, we used the IgDiscover *clonotypes* module outputs containing one representative sequence for each clonotype as our reference database of SARS‐CoV‐2 specific sequences. We queried clonal relatives of this database in bulk IgG/M HTS dataset using the *clonoquery* function. Total number of bulk BCR sequences, clonotypes, and clonal size identified in bulk sequencing libraries per individual are shown in Supplementary table 2 (S3).

### Clonotype diversity estimation

The estimation of sample coverage interpolation and extrapolation was calculated using the vegan R package based on the clonal sizes of the merged datasets. The species richness estimation was calculated using the Chao1 and ACE1 algorithms with the vegan R package.^70^ The estimation was performed per individual study participant.^70^ To normalise for sequencing depth, clonal sizes were subsampled 100× to the lowest number of sequences per individual. The average per individual was used as input to estimate Hill numbers, and the Chao1 index was used for plotting.^70^

### Assessment of SARS‐CoV‐2 specific memory T cell responses

Antigen‐specific T cells were assessed by peptide stimulation followed by intracellular cytokine staining (ICS) as previously described.^58^ Briefly, PBMCs were thawed as described for memory B‐cell assessment. One to two million PBMCs were stimulated overnight at 37°C, 5% CO_2_, with either 2 mg mL^−1^ overlapping peptides (15‐mers overlapping by 11) covering the SARS‐CoV‐2 Spike protein (JPT), 1 mg mL^−1^ Staphylococcal Enterotoxin B (Sigma‐Aldrich) as a positive control, or R10 with 0.8% DMSO as a negative control. Brefeldin A (Thermo Fisher) was added to all conditions for a final concentration of 10 mg mL^−1^. Following stimulation, cells were washed twice with PBS, stained first with Live/Dead Fixable Blue or Live/Dead Fixable Aqua viability dyes (Invitrogen) for 5 min at 4°C followed by anti‐human CCR7‐BV421 (G043H7; Biolegend), CD8a^−^BV711 (RPA‐T8, Biolegend), CD4^−^PE‐Cy55 (S3.5; Invitrogen), CD45RA‐BV650 (5H9; Biolegend) for 20 min at 4°C. Following surface staining, cells were washed twice with PBS and permeabilised using the BD Cytofix/Cytoperm kit (BD Biosciences) for 20 min at RT, washed twice and stained intracellularly with anti‐human IL‐21‐AF647 (3A3‐N2.1; BD Biosciences), IL‐13‐PE (JES10‐5A2; BD Biosciences), IL‐2‐BV605 (MQ1‐17H12; BD Biosciences), IL‐17A‐BV785 (BL168; Biolegend), CD69^−^ECD (TP1.55.3; Beckman‐Coulter), CD3^−^APC‐Cy7 (SP34.2; BD Biosciences), IFNg‐AF700 (B27; Biolegend), and TNFa‐AF488 (Mab11; BD Biosciences) for 20 min at 4°C. Following ICS, cells were washed with BD 1× Fix/Perm buffer, resuspended in 1% PFA and acquired on an LSRFortessa flow cytometer (BD Biosciences). Data was analysed using Flowjo version 10 (FlowJo Inc.). Representative gatings are shown in Supplementary figure 14. The value for the unstimulated control was subtracted from the peptide stimulated condition and the result used as the final readout.

### Statistical methods

Group comparisons were performed using the Kruskal–Wallis test with Dunn's multiple comparisons post hoc with *P* value adjustment using Graphpad Prism v. 10.0 default settings (where using R, *P*‐value was adjusted for multiple comparisons using the Benjamini & Hochberg method for false‐discovery rate) when testing differences between three or more groups. The Mann–Whitney *U* test was used when comparing only two groups, or the Wilcoxon signed‐rank test for paired samples. Two‐tailed *P*‐value was used in all statistical tests comparing study groups. Spearman correlation with two‐tailed *P*‐value was used to analyse correlations between different parameters. Nonparametric tests were used as the low sample size did not permit the assumption of normal distribution.

Statistical analyses were performed using Graphpad Prism version 10.0 (La Jolla California, USA) or R (4.4.0), unless otherwise indicated in methods sections above. A *P*‐value < 0.05 was considered significant for all comparisons.

Multivariable linear regression was used to assess the association between transitional B (TrB) cell frequency and vaccine‐induced antibody responses. Log‐transformed antibody titres ED50 values were used to better align with linear regression assumptions. Multicollinearity among predictors was evaluated using variance inflation factors (VIF), with no evidence of collinearity observed. Given the small sample size and residual variability, heteroskedasticity‐consistent HC3 robust standard errors were applied.

The final model was specified as:

$$\log\left(\mathrm{ED}50\right)=\mathrm{tim}e_{\text{days}}+{\text{immunosupp}}_{\mathrm{bin}}+\mathrm{TrB}$$

where time_days represents the time elapsed since transplantation (in days), immunosupp is a binary indicator of immunosuppressive treatment, and TrB denotes the percentage of transitional B cells among total CD19^+^ B cells.

## Author contributions

**Fredrika Hellgren:** Conceptualization; investigation; methodology; formal analysis; data curation; visualization; writing – original draft; writing – review and editing. **Rodrigo Arcoverde Cerveira:** Conceptualization; investigation; methodology; formal analysis; data curation; visualization; writing – review and editing. **Gustaf Lindgren:** Investigation; resources; writing – review and editing. **Puran Chen:** Investigation; writing – review and editing. **Klara Lenart:** Investigation; writing – review and editing. **Sebastian Ols:** Investigation; writing – review and editing. **Alberto Cagigi:** Investigation; methodology; writing – review and editing. **Davide Valentini:** Investigation; resources; writing – review and editing. **Mireia Rocavert Barranco:** Investigation; writing – review and editing. **Evangelin Shaloom Vitus:** Investigation; methodology; writing – review and editing. **Martin Corcoran:** Resources; writing – review and editing. **Yong‐Dae Gwon:** Investigation; writing – review and editing. **Mattias NE Forsell:** Investigation; writing – review and editing. **Magnus Evander:** Resources; methodology; writing – review and editing. COVAXID Study Group: Investigation; resources. **Peter Bergman:** Writing – review and editing. **Marcus Buggert:** Conceptualization; methodology; writing – review and editing. **Hans‐Gustaf Ljunggren:** Conceptualization; funding acquisition; writing – review and editing. **Soo Aleman:** Conceptualization; funding acquisition; project administration; writing – review and editing. **Gunilla B Karlsson Hedestam:** Conceptualization; funding acquisition; supervision; writing – review and editing. **Andreas Björklund:** Methodology; resources; writing – review and editing. **Anna Nordlander:** Investigation; resources; writing – review and editing. **Per Ljungman:** Resources; writing – review and editing. **Stephan Mielke:** Conceptualization; supervision; funding acquisition; project administration; writing – review and editing. **Karin Loré:** Conceptualization; supervision; funding acquisition; project administration; writing – review and editing.

## Conflict of interests

M.B. is a consultant and has received honoraria from Oxford Immunotec, MSD, BMS, Pfizer and Mabtech. S.A. has received honoraria for lectures and educational events, not related to this work, from Gilead, AbbVie, MSD, Biogen, and reports grants from Gilead and AbbVie. All other authors declare that they have no competing interests. This study has been funded by grants from Knut and Alice Wallenberg Foundation (through SciLifeLab and Karolinska Institutet grant VC‐2021‐0017), the Swedish Research Council (Vetenskapsrådet) (Grants 2019–01036, 2020–05929, and 2023–02396 to KL, Grant 2017–00968 to GKH), Cancerfonden (211 728 Pj), as well as intramural graduate student fellowships from Karolinska Institutet. The COVAXID phase IV clinical trial has been funded by grants from the Knut and Alice Wallenberg Foundation (through SciLifeLab) VC‐2021‐0018 and Swedish Research Council (Vetenskapsrådet) 2021–04779. For equipment and resources, see ‘Acknowledgements’.

## Code availability statement

All the code used for sequencing analysis will be publicly available on a Github repository (github.com/Lore‐Lab‐Vaccine‐Immunology/CAST_2021) upon publication.

## Supporting information


Supplementary figure 1–16

Supplementary table 1

Supplementary table 2

## Data Availability

The sequencing BCR data are deposited in the Swedish National Data Service (SND): https://doi.org/10.48723/bbag‐t656. Characteristics of the data are reported in Supplementary table 2 (S3). The data set contains sensitive personal data. Access to data can be requested via the SND research data catalogue. The request needs to be approved by the research principal and might need to undergo a confidentiality assessment before access can be released.
