# Supplementary material for: The impact of B‐cell reconstitution on mRNA vaccine responses in allogeneic stem cell transplant recipients
Source: Clin Transl Immunology. 2026 Feb 11;15(2):e70077. doi: 10.1002/cti2.70077 (PMC12892404; doi:10.1002/cti2.70077)
Supplement: Supplementary file 1 — Supplementary figure 1–16 [file CTI2-15-e70077-s001.docx]

**Supplementary information to:**

**The Impact of B Cell Reconstitution on mRNA Vaccine Responses in Allogeneic Stem Cell Transplant Recipients**

Fredrika Hellgren^1,2^, Rodrigo Arcoverde Cerveira^1,2^, Gustaf Lindgren^3,4,5^, Puran Chen^6^, Klara Lenart^1,2,13^, Sebastian Ols^1,2,14^, Alberto Cagigi^1,2,15^, Davide Valentini^3,7^, Mireia Rocavert Barranco^1,2^, Evangelin Shaloom Vitus^1,2^, Martin Corcoran^8^, Yong-Dae Gwon^9^, Mattias N.E. Forsell^9^, Magnus Evander^9^, COVAXID study group, Peter Bergman^10,11^, Marcus Buggert^6^, Hans-Gustaf Ljunggren^5,6^, Soo Aleman^6,12^, Gunilla B. Karlsson Hedestam^8^, Andreas Björklund^3,4,5,6^, Anna Nordlander^3,4,5,7,12,^ Per Ljungman^3,4,6^, Stephan Mielke*^3,4,5,7^, Karin Loré*^1,2^

*^1^Division of Immunology and Respiratory Medicine, Department of Medicine Solna, Karolinska Institutet, Stockholm, Sweden & Karolinska University Hospital, Stockholm, Sweden.*

*^2^Center for Molecular Medicine, Karolinska Institutet, Stockholm, Sweden.*

*^3^Department of Cellular Therapy and Allogeneic Stem Cell Transplantation (CAST), Karolinska University Hospital Huddinge, Stockholm, Sweden*

*^4^Karolinska Comprehensive Cancer Center, Karolinska Institutet and University Hospital, Stockholm, Sweden*

*^5^Karolinska ATMP Center, Karolinska Institutet and University Hospital, Stockholm, Sweden*

*^6^Department of Medicine Huddinge, Karolinska Institutet, Stockholm, Sweden*

*^7^Department of Laboratory Medicine, Clinical Immunology, Karolinska Institutet, Stockholm, Sweden*

*^8^Department of Microbiology, Tumor and Cell Biology, Karolinska Institutet, Stockholm, Sweden.*

*^9^Department of Clinical Microbiology, Umeå University, Umeå, Sweden*

*^10^ Department of Laboratory Medicine, Biomolecular and Cellular Medicine (BMC), Karolinska Institutet, Stockholm, Sweden*

*^11^Department of Clinical Immunology and Transfusion Medicine, Karolinska University Hospital, Stockholm, Sweden*

*^12^Department of Infectious Diseases, Karolinska University Hospital, Stockholm, Sweden*

**equal contribution*

*13: Current affiliation: Rockefeller University, New York, USA.*

*14: Current affiliation: University of Washington, Seattle, USA.*

*15: Current affiliation: International Vaccine Institute Europe Regional Office, Stockholm, Sweden*

*Corresponding authors: Stephan Mielke (*[Stephan.Mielke@ki.se](mailto:Stephan.Mielke@ki.se)*) and Karin Loré (*[Karin.lore@ki.se](mailto:Karin.lore@ki.se)*)*

**
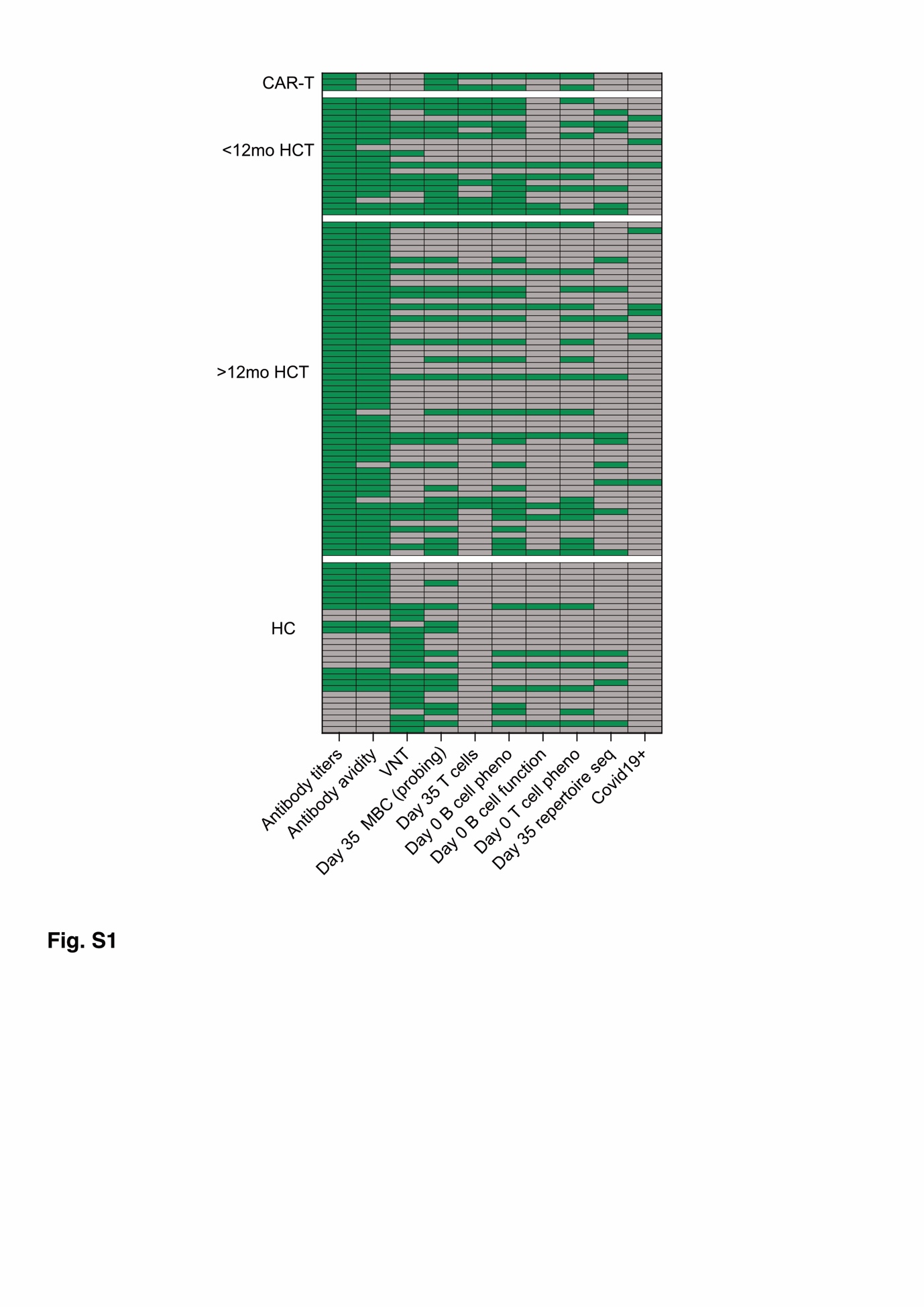
Fig. S1. Summary of available data per study participant and subgroup for key data subsets**. Each row represents one study participant. Each column represents one data category or group of experiments. Green = data available (not including exclusions done due to technical reasons, see respective figure legends).

**
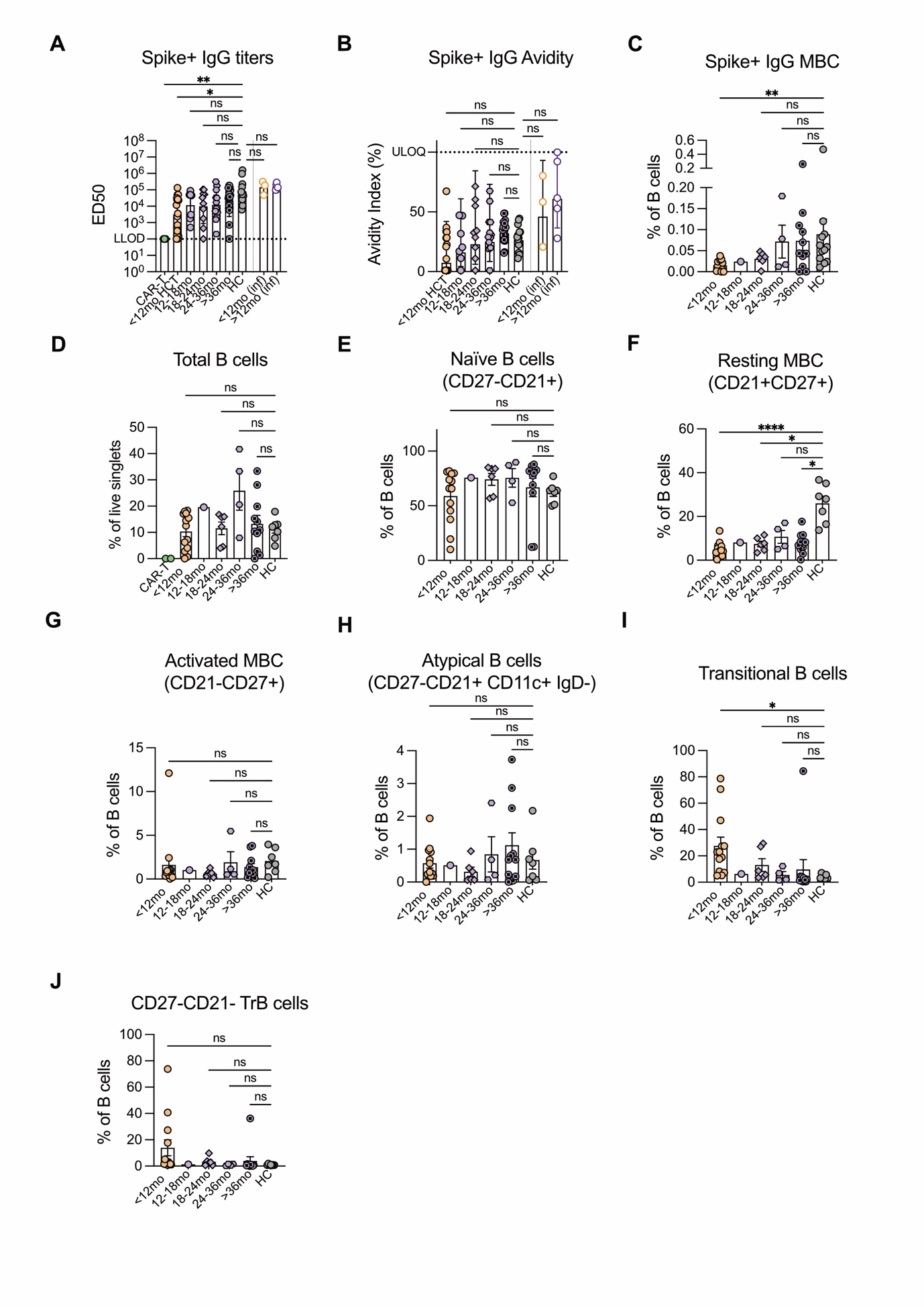
Fig S2:** **Vaccine immune responses and baseline characteristics using further time-dependent stratification of the >12mo alloHCT group, key data**. **A:** Day 35 Spike-binding plasma IgG titers quantified by ELISA, expressed as half-maximal binding dilution reciprocal (ED50). **B:** Day 35 Spike-binding plasma IgG avidity quantified by chaotropic avidity ELISA, expressed as % binding remaining after chaotropic treatment compared to PBS-treated control. Samples with binding <LLOD after chaotropic treatment were assigned an arbitrary value of 1%. **C:** Day 35 Specific IgG+ B cells quantified by fluorescent probe staining and flow cytometry, expressed as percentage of total B cells. D-J: Day 0 B cell subpopulations measured by flow cytometry. **D:** Total CD19+ B cells, expressed as % of total live single cells. **E:** CD27-CD21+ naïve B cells, expressed as % of total CD19+ B cells. **F:** CD27+CD21+ resting memory B cells, expressed as % of total CD19+ B cells. **G:** CD27+CD21- activated memory B cells, expressed as % of total CD19+ B cells. **H:** CD27-CD21- IgD-CD11c+ atypical B cells, expressed as % of total CD19+ B cells. **I:** CD24hiCD38hi transitional B cells, expressed as % of total CD19+ B cells. **J:** CD27-CD21- transitional B cells, expressed as % of total CD19+ B cells. Error bars represent geometric mean +/- geometric SD (A) or mean +/- SEM (B-J).


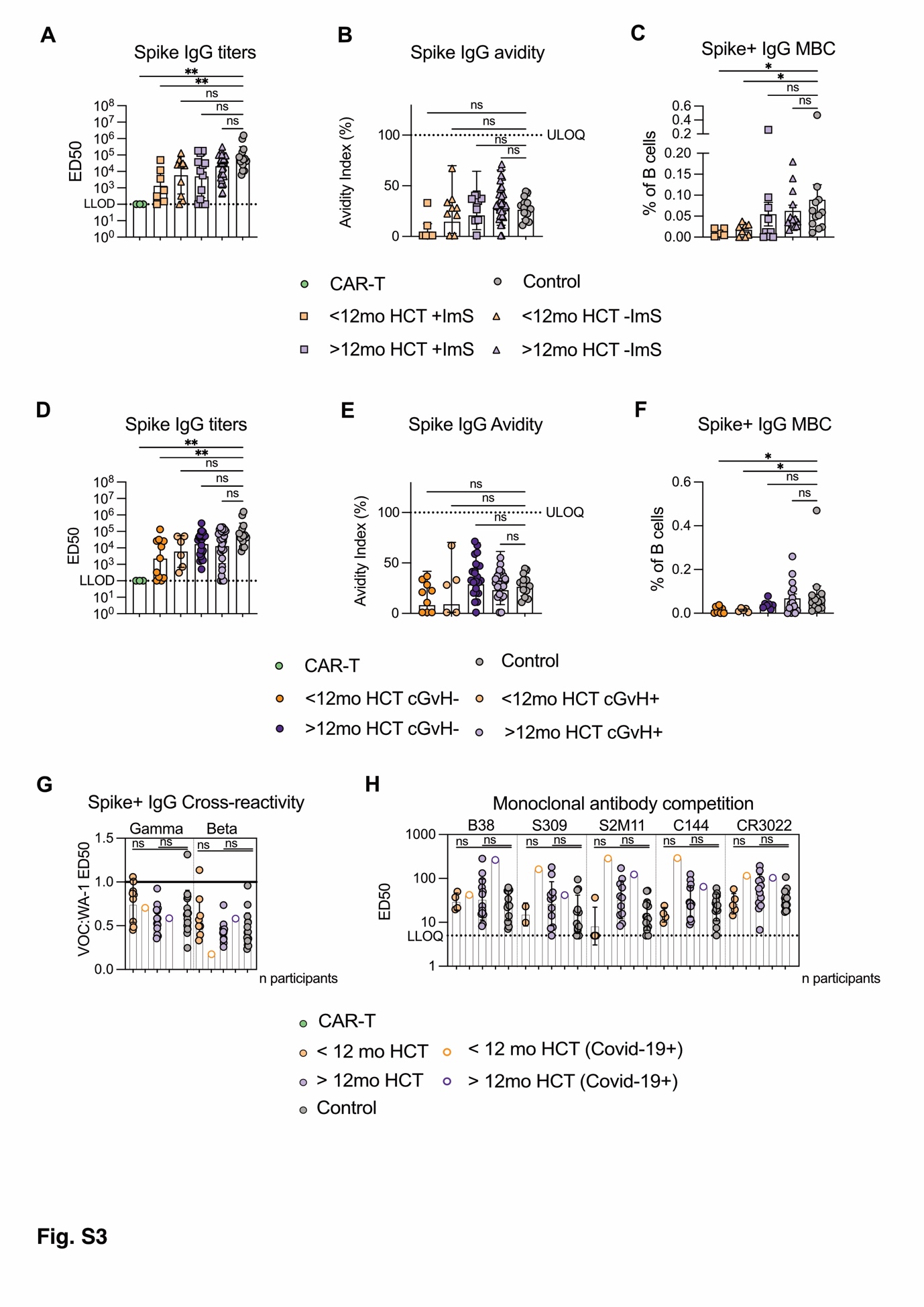
**Fig. S3: Vaccine immune responses stratified by ongoing pharmacological immunosuppression (A-C), and presence of chronic graft-versus-host disease (cGVHD) (D-F). Antibody cross-reactivity & epitope mapping (G-H).** **A, C**: Spike-binding antibody titers quantified by ELISA. **B, E**: Spike-binding IgG avidity measured by chaotropic ELISA using 1.5M NaSCN. C,F: Spike-binding IgG+ B cells measured by fluorescent protein probe staining and flow cytometry analysis. **G:** Cross-reactivity of Spike-binding serum IgG to viral variant Spikes measured by ELISA, reported as the ratio of variant-binding ED50 to ancestral strain S binding ED50. **H:** Serum levels of antibody binding to specific epitopes of the S protein, assessed by degree of competition against previously characterized monoclonal antibodies. Samples showing poor competition with mAbs were excluded from analysis as an ED50 could not be reliably computed. All group statistical comparisons by Kruskal-Wallis test with Dunn’s post hoc test comparing alloHCT and CAR-T study groups to healthy controls, unless otherwise specified. Where present, error bars indicate geometric mean +/- geometric SD.
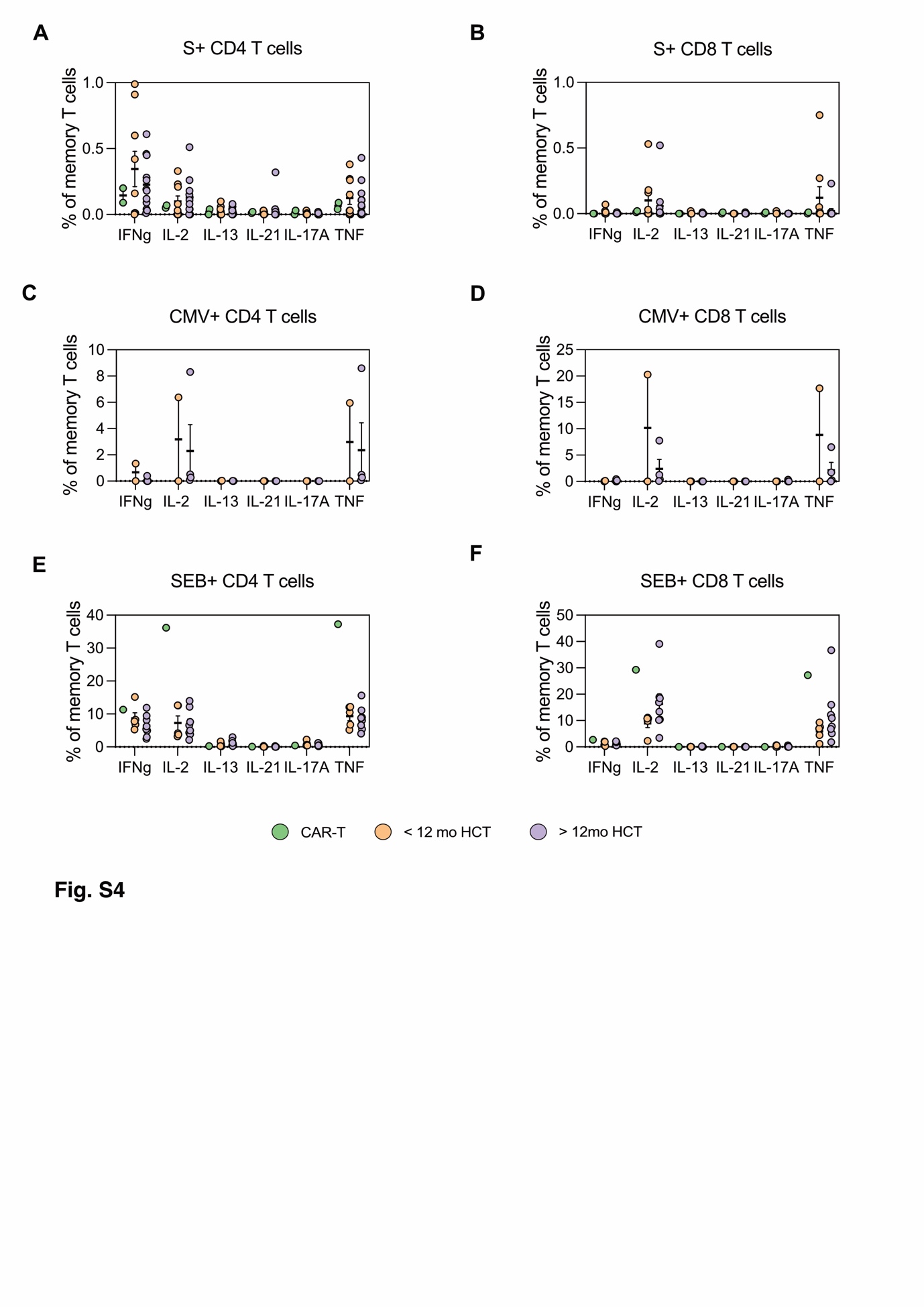


**Fig S4: Antigen-specific T cell responses.** A-B: Cytokine-producing CD4 **(A)** and CD8 **(B)** memory T cells after ex vivo restimulation with overlapping peptides representing the SARS-CoV-2 Spike protein. **C-D:** Cytokine-producing CD4 **(C)** and CD8 **(D)** memory T cells after ex vivo restimulation with overlapping peptides representing selected CMV antigens. **E-F:** Cytokine-producing CD4 **(E)** and CD8 **(F)** memory T cells after ex vivo restimulation with Staphylococcal Enterotoxin B (SEB). Data displayed as percentage of total CD4 **(A, C, E)** or CD8 **(B, D, F)** memory T cells, respectively. All data pertain to Day 35 (post prime-boost mRNA vaccination). N reported in Table 1 refer to assessment of Spike+ CD4/CD8 T cells, which were prioritized for analysis. CMV and SEB stimulated conditions were additionally conducted on all samples with sufficient cells. Error bars indicate mean +/- SEM. No statistical test performed due to lack of healthy control group and generally small sample sizes.

**
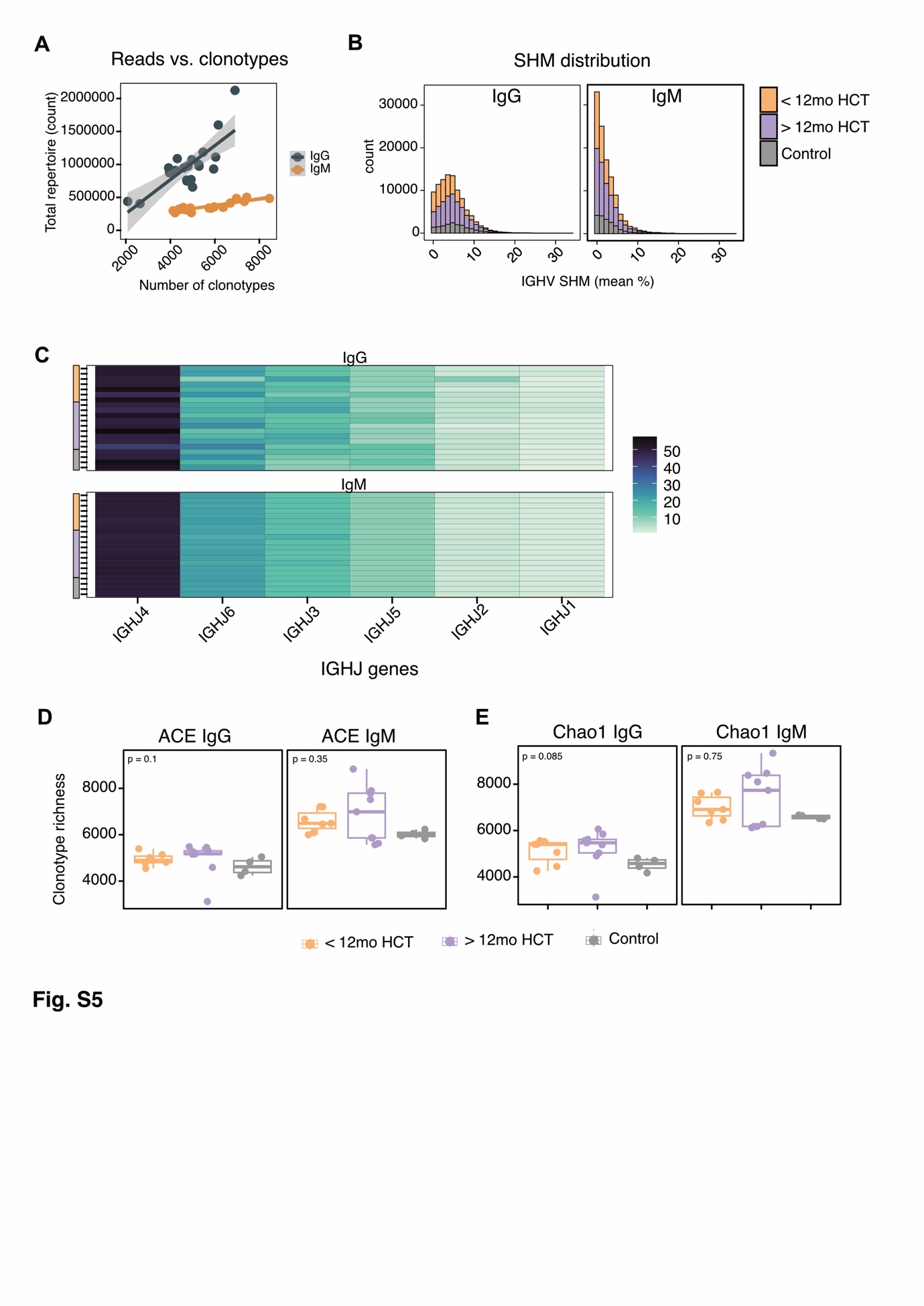
Fig. S5: Characteristics of bulk IgG/IgM repertoire sequencing data.**

**A:** Total number of retrieved reads (UMI corrected) and number of clonotypes, divided by IgG and IgM**. B:** Distribution of V gene SHM (%) per identified clonotype. **C:** Frequency of J gene usage per identified clonotype, expressed as percentage of full repertoire. **D-E:** Total clonotype diversity per study participant repertoire sequenced, expressed as either ACE (D) or Chao1 (E) indexes. Group comparison by Kruskal-Wallis test. N = 20 unless otherwise specified. All data pertain to day 35 (post prime-boost mRNA vaccination).

**
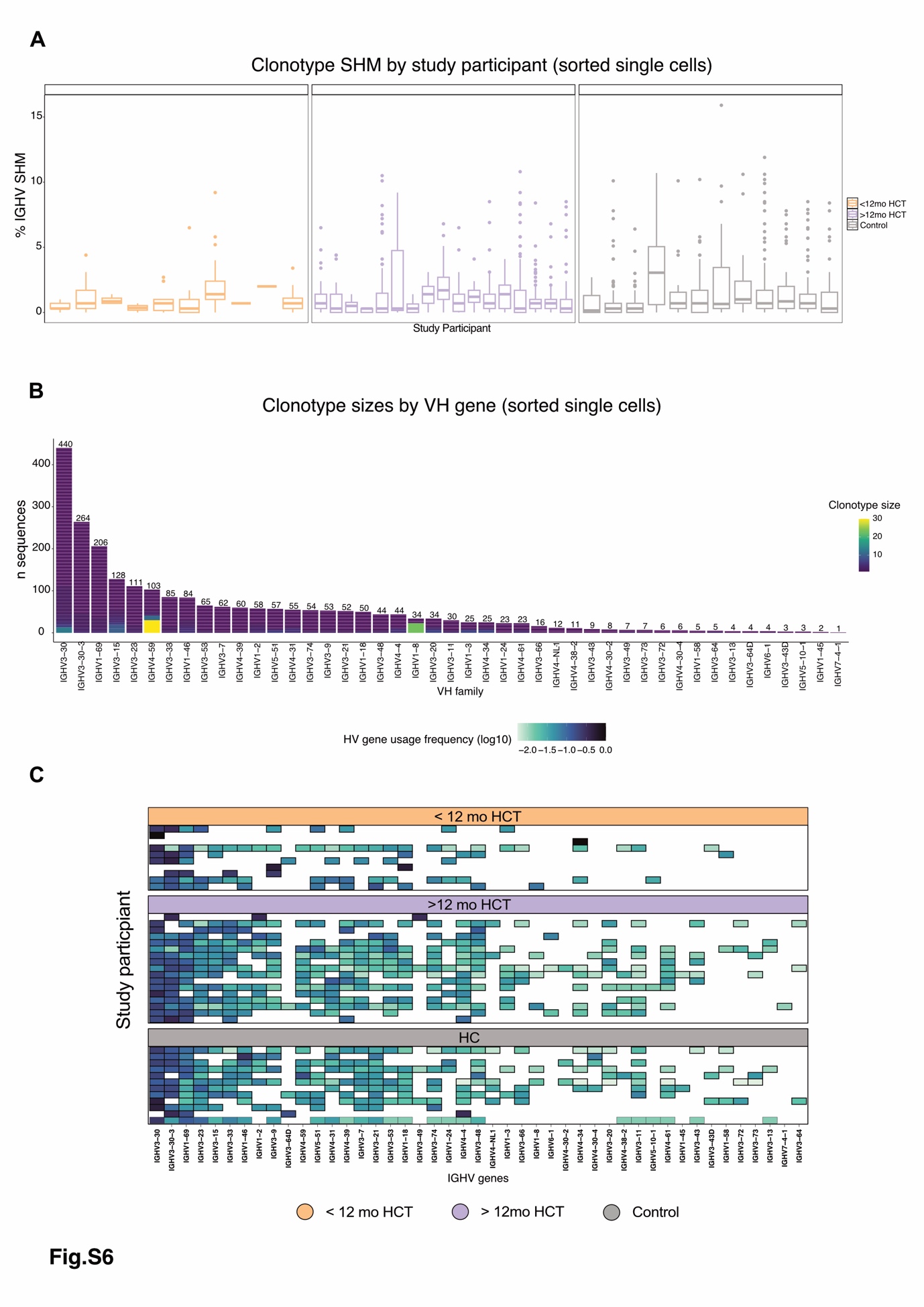
Fig. S6: Characteristics of sorted Spike-binding IgG BCR IGH.**

**A:** Percentage IGHV somatic hypermutation (SHM) accumulated, quantified through alignment to the OGRDB immunoglobulin germline database and SHM calculation using IgBlast implemented in IgDiscover software. SHM per identified clonotype is shown. Clonotypes were defined by identical heavy chain V-J gene pairing and 80% CDRH3 identity. **B:** VH gene usage across Spike+ BCRs obtained through single-cell sorting and sequencing, irrespective of study group. Each bar represents one VH family. Bars represent total number of sequences, stacks represent identified clonotypes. Color scale denotes clonotype size (n sequences). **C:** VH gene usage per Spike+ clonotype and study participant sequenced. Each row represents one study participant. Color scale denotes proportion of total identified S+ clonotypes per individual participant that aligned to a particular IGHV family. All BCR sequences were aligned to the OGRDB immunoglobulin germline database using IgDiscover software. All data pertain to day 35 (post prime-boost mRNA vaccination). N study participants = 39 (27 alloHCT, 12 HC).

**
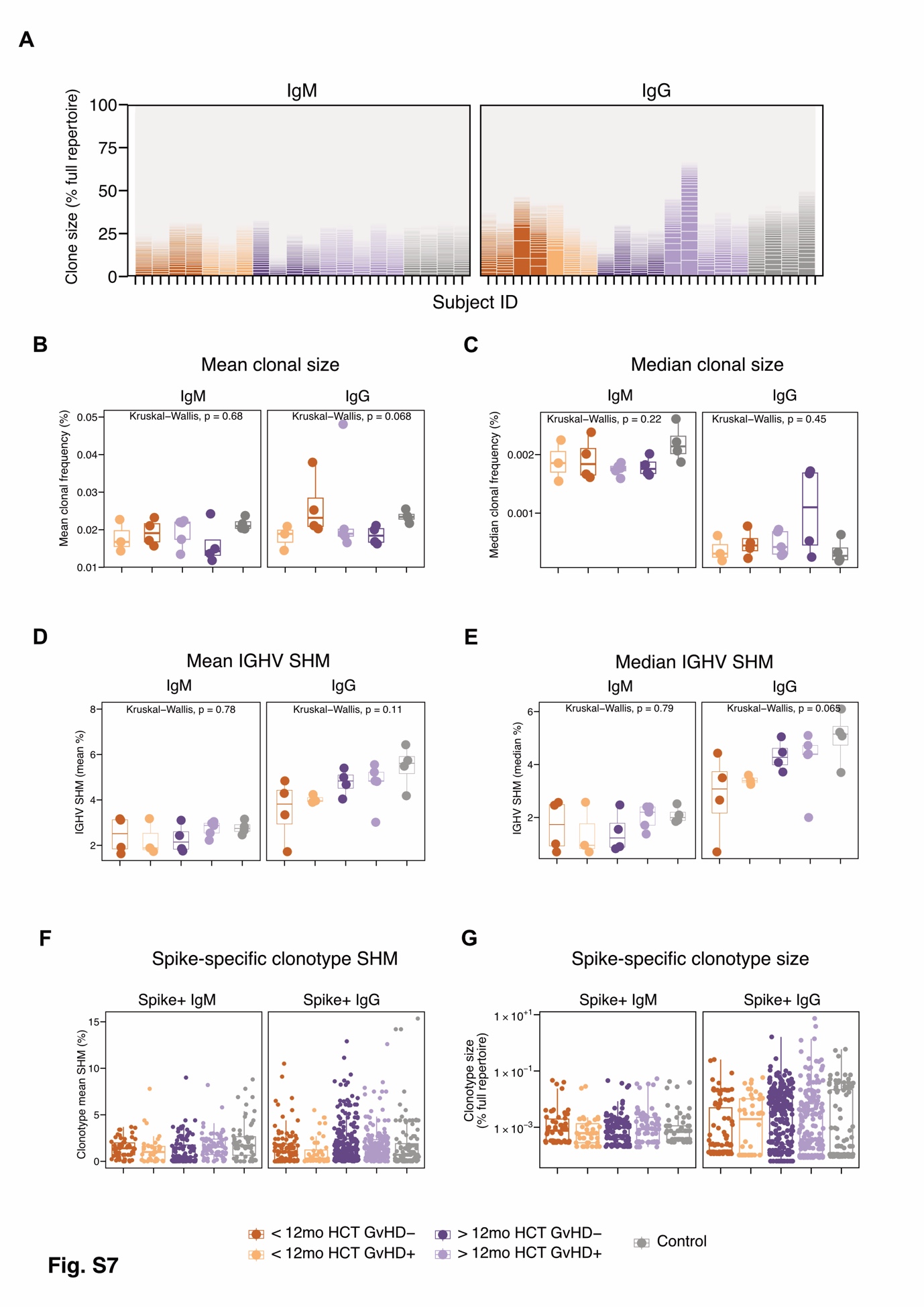
Fig. S7: Additional repertoire sequencing analysis subdivided by presence and absence of cGVHD.**

**A:** Proportional distribution of IgM (left) and IgG (right) BCR clonotypes identified per full repertoire sequenced, divided by time post HSCT and cGVHD status. Each column represents one repertoire and study subject. Each line/block represents one clone. Clonotypes are defined by identical heavy chain V-J gene pairing and 80% HCDR3 identity. Y axis shows each clonotype as a percentage of the total number of clonotypes identified per repertoire and isotype. **B-C:** Mean (B) and median (C) clonotype frequency, expressed as percentage of total repertoire. **D-E:** Mean (D) and median (E) IGHV somatic hypermutation (SHM) accumulated, quantified through alignment to the OGRDB immunoglobulin germline database and SHM calculation using IgDiscover software. **F-G:** Mean SHM (F) and clonal size as percentage of full repertoire (G) of individual clonotypes identified as Spike+ by query of bulk IgG/M databases using probe-sorted IgG BCRs as reference. No statistical tests shown for F-G. Data was analyzed using IgDiscover version 0.15.1 and open-source R packages. N = 20 (A-E) or 19 (F-G, one HCT recipient omitted due to Covid-19 infection experience). All data pertain to day 35.

**
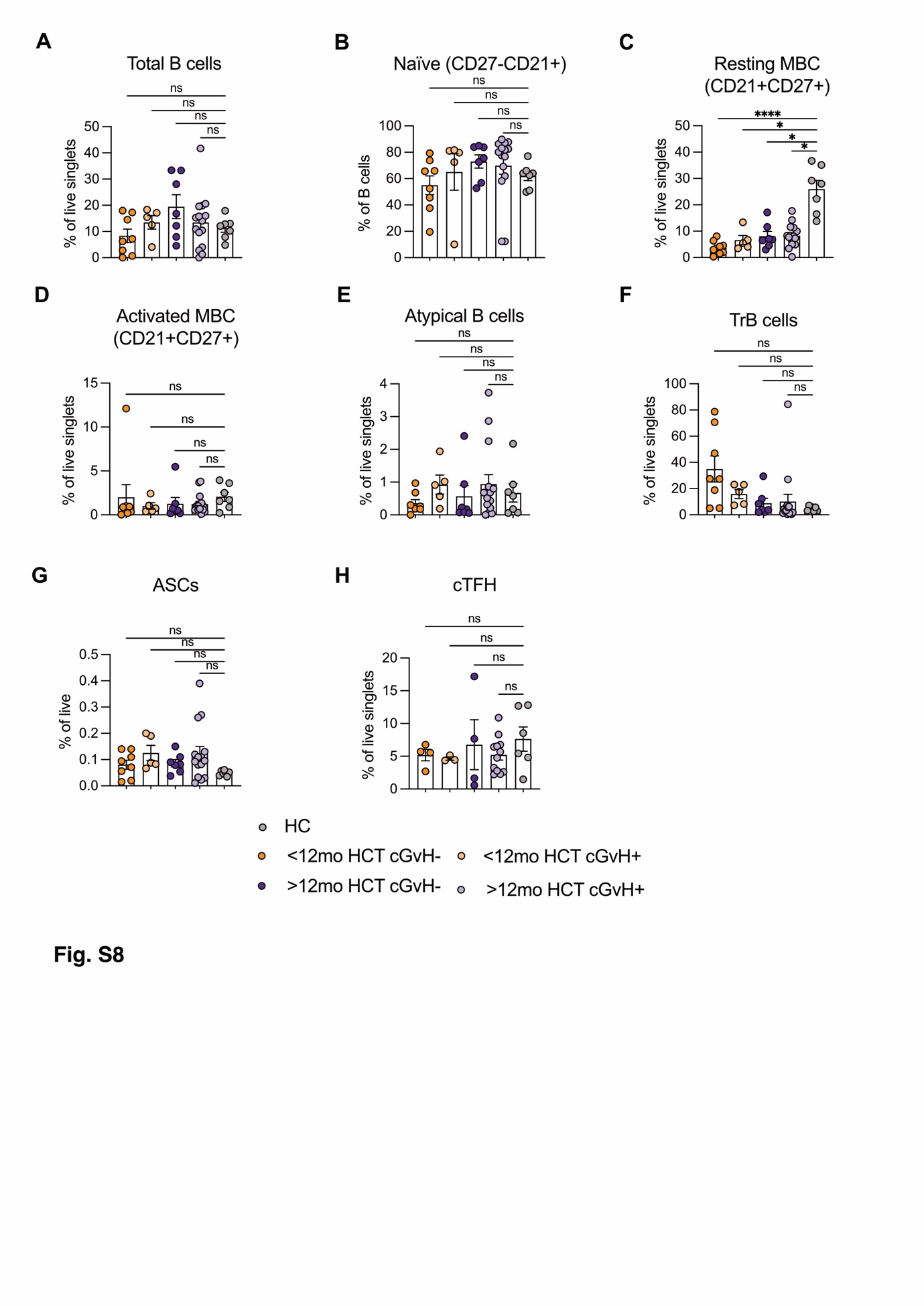
Fig. S8: Day 0 B cell subpopulations and cTFH stratified by presence of chronic GvH (cGVH).**

**A:** Total CD19+ B cells shown as percentage of total live single cells. **B - E:** Quantitation of naïve B cells **(B)**, resting memory B cells **(C)**, activated memory B cells **(D)**, and atypical B cells **(E)**, shown as percentages of total CD19+ B cells. **F:** Quantitation of TrB cells, shown as proportion of total CD19+ B cells. **G:** Quantitation of CD20lo-CD38hiCD27hi plasmablasts, shown as proportion of total live singlets. **H:** Total cTfh cells in periopheral blood, expressed as percentage of total CD4+ T cells. Where present, error bars indicate mean +/- SEM.

**
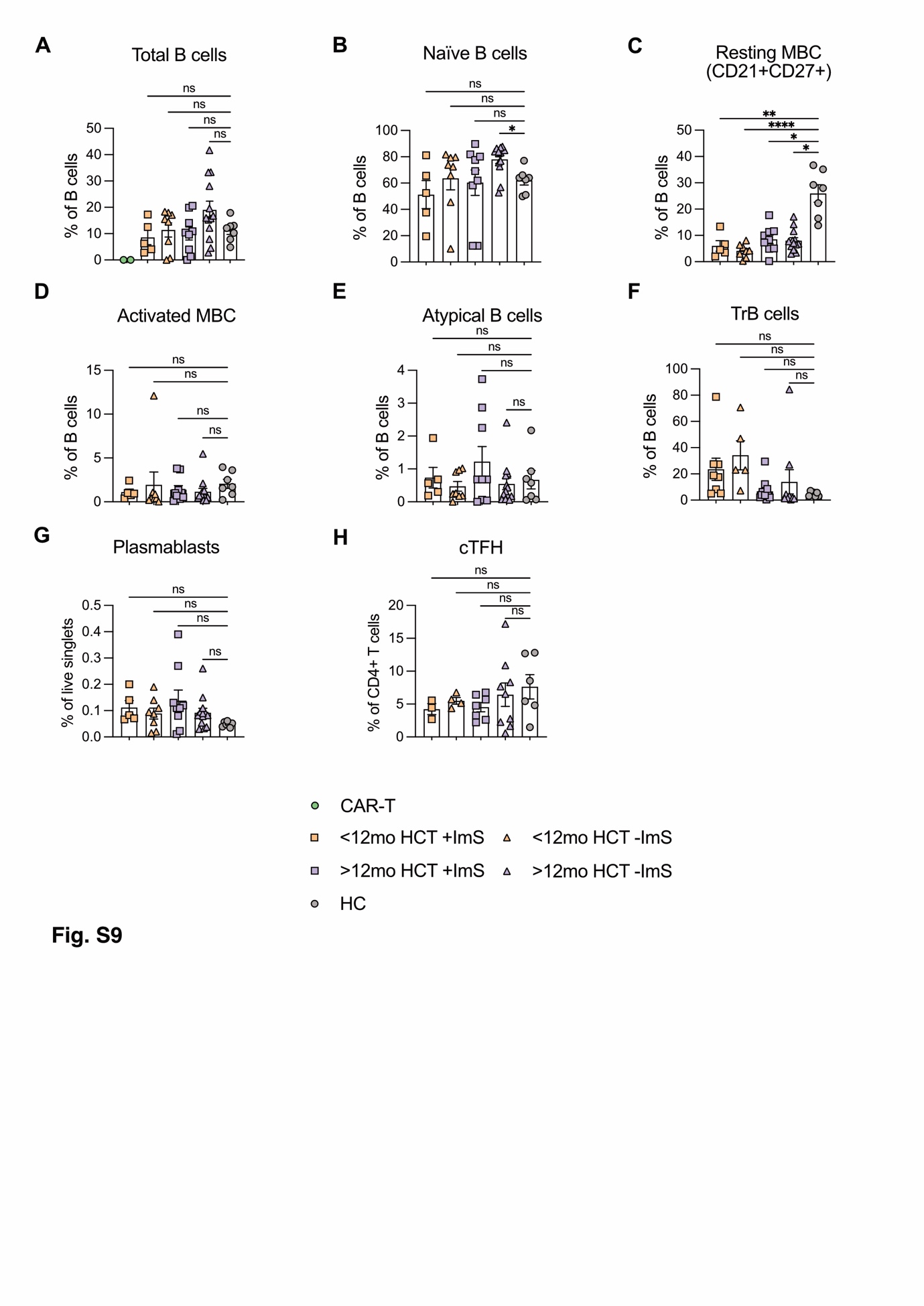
Fig. S9: Day 0 B cell subpopulations and cTFH stratified by presence of immunosuppressive treatment. A:** Total CD19+ B cells shown as percentage of total live single cells. **B - E:** Quantitation of naïve B cells **(B)**, resting memory B cells **(C)**, activated memory B cells **(D)**, and atypical B cells **(E)**, shown as percentages of total CD19+ B cells. **F:** Quantitation of TrB cells, shown as proportion of total CD19+ B cells. **G:** Quantitation of CD20lo-CD38hiCD27hi plasmablasts, shown as proportion of total live singlets. **H:** Total cTfh cells in peripheral blood, expressed as percentage of total CD4+ T cells. Where present, error bars indicate mean +/- SEM.

**
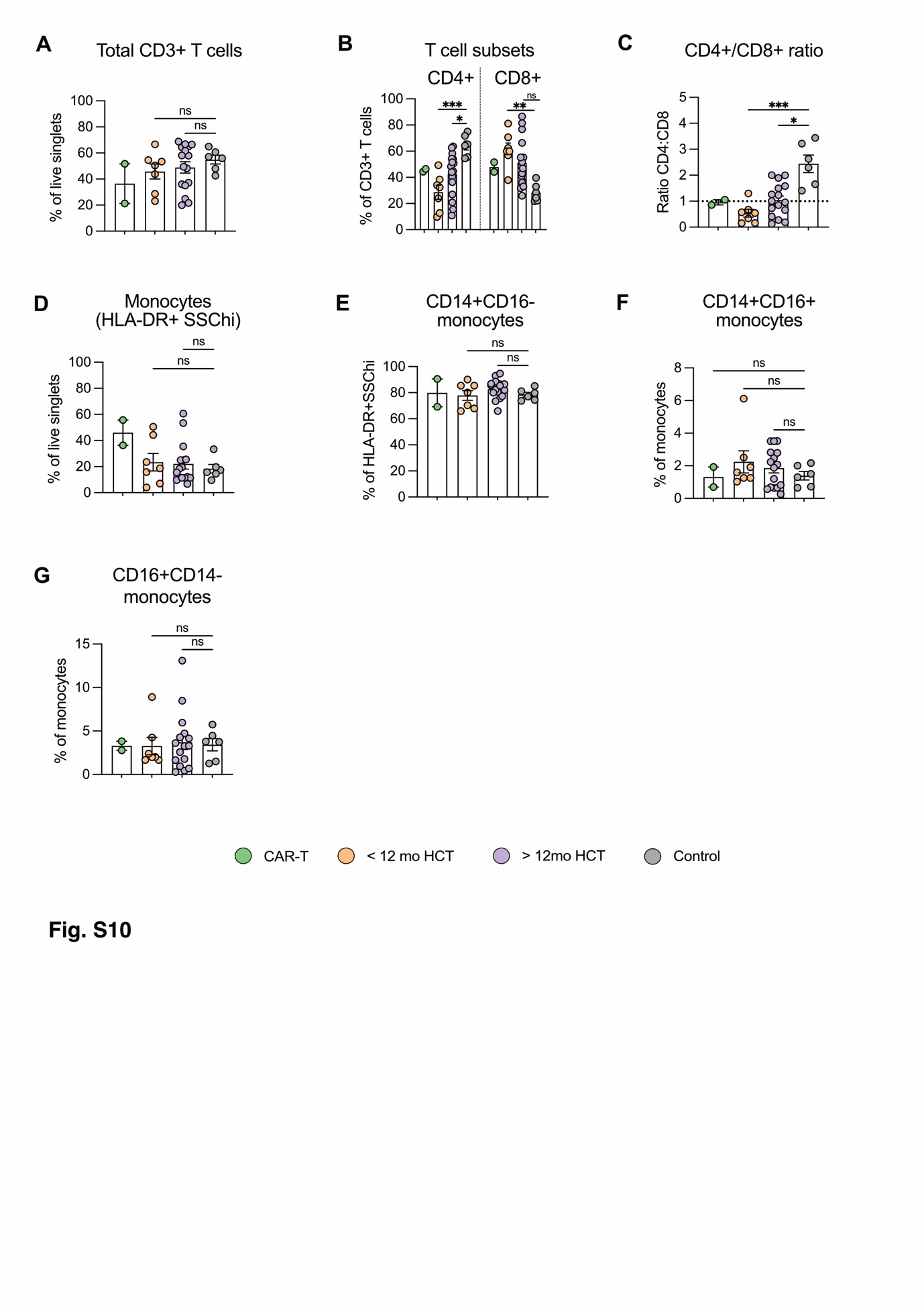
Fig. S10:** **T-cell and innate immune populations at Day 0.** **A:** Total CD3 T cells expressed as percentage of total live single cells. **B-C:** Total CD4+ (B) and CD8+ (B) T cells expressed a percentage of total CD3+ T cells. **D:** Monocytes, identified as CD3-Lineage-/lo, SSChi cells, expressed as percentage of total live single cells. **E-G:** CD14+CD16- classical monocytes (E), CD14+CD16+ intermediate monocytes (F), and CD14-CD16+ non classical monocytes (G), expressed as percentages of total monocytes. All analyses pertain to Day 0 (pre-vaccination). Where present, error bars indicate mean +/- SEM.


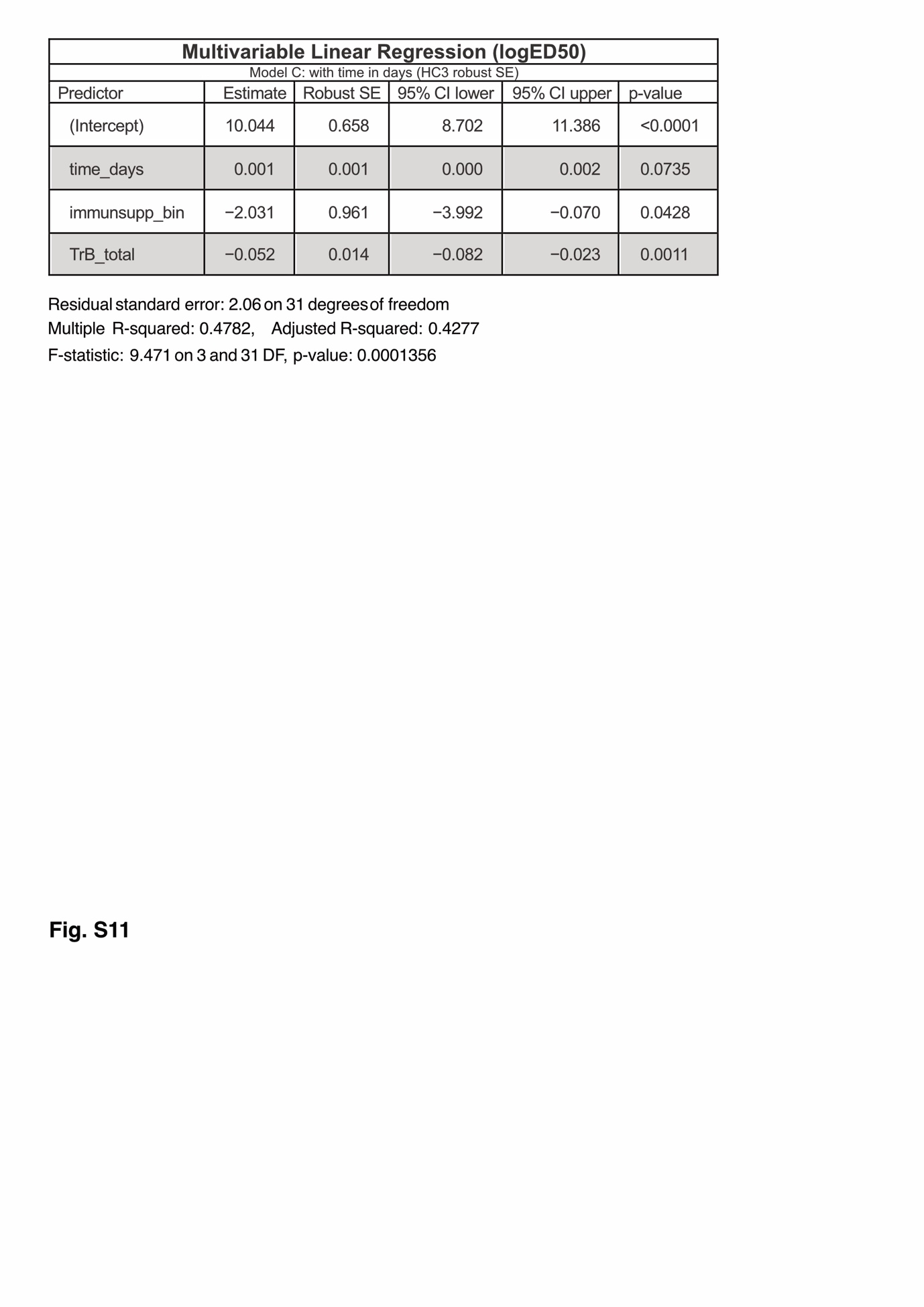


**Fig. S11: Multivariable linear analysis assessing association of baseline transitional B cells in predicting log(ED50) spike-binding antibody titers.** The model employed was as follows: *log (spike-binding antibody ED50) = time post alloHCT (continuous, n days) + immunosuppression (binary yes/no) + TrB (continuous, % of total CD19+ B cells).* Data from 35 alloHCT recipients were included in the analysis (all individuals for whom day 0 B cell phenotyping data was available). Note: Because the outcome was analyzed on the log scale, regression coefficients of the following regressions should be interpreted in relative (multiplicative) terms. A coefficient corresponds to an approximate percentage change in antibody titers associated with a one-unit increase in the predictor, holding other variables constant. For example, a coefficient of −0.05 indicates an approximate 5% decrease in the antibody response per one-unit increase in the predictor, rather than an absolute decrease in ED50. (e−0.05−1)×100≈−4.9%

**
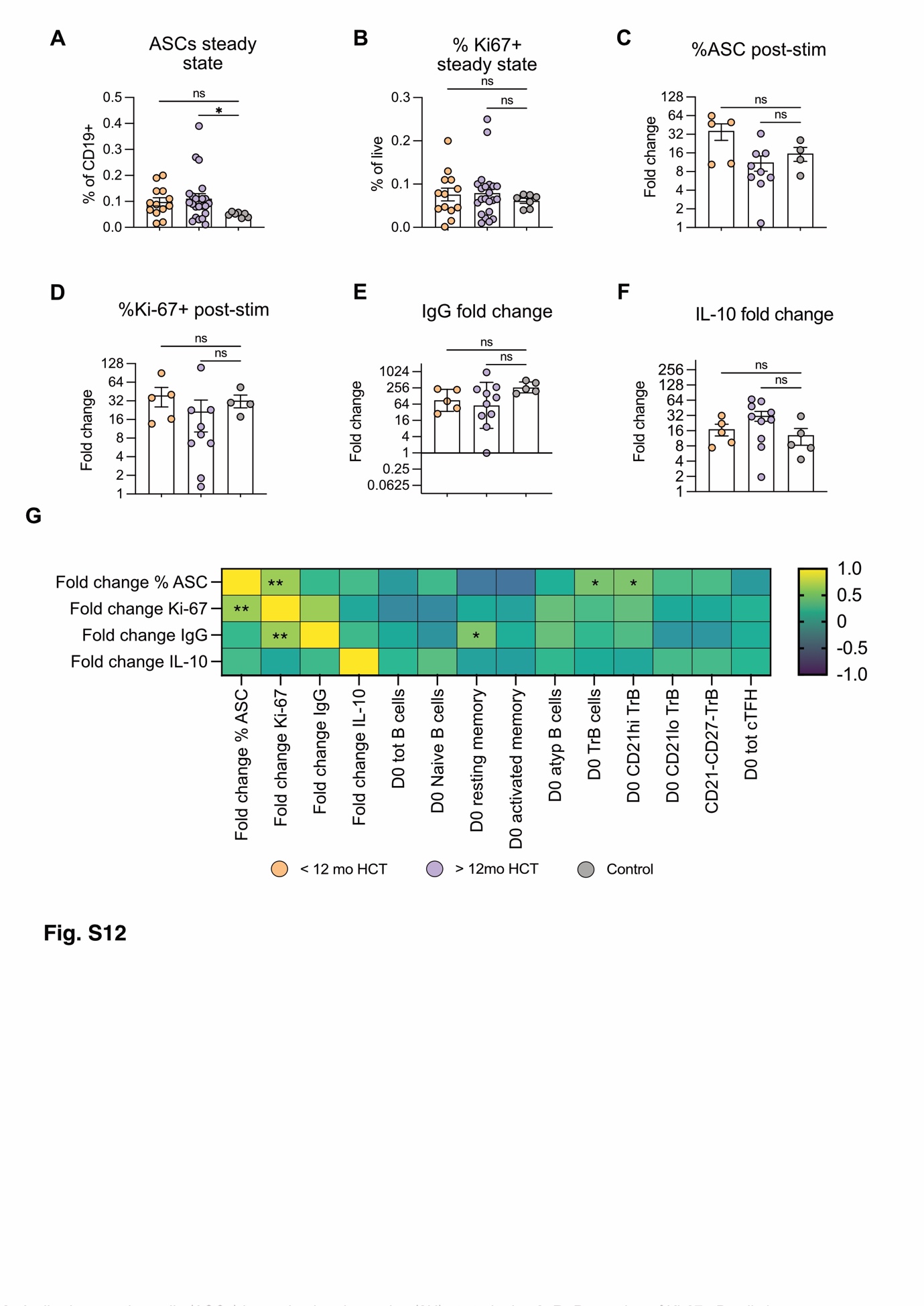
Fig. S12: Additional data Day 0 B cell ex vivo activation. A:** Proportion of antibody-secreting cells (ASCs) in unstimulated samples (0H) at study day 0. **B:** Proportion of Ki-67+ B cells in unstimulated samples (0H) at study day 0. **C-D:** Fold change in **%**ASCs (C) and Ki-67+ B cells (D) in 6-day cultures of PBMC in the presence (stim) or absence (unstim) of mouse-anti-human IgM Fab(2) fragments, CpGB, soluble CD40L and IL-21. **E:** Fold change in IgG supernatant concentration between stimulated samples and unstimulated controls. **F:** Fold change in IL-10 supernatant concentration between stimulated samples and unstimulated controls. **G:** Non-parametric Spearman correlations of fold changes in ASC, Ki-67, IL-10 and IgG (calculated between stimulated samples and unstimulated control wells) to proportions of different B cell populations at Study Day 0 as shown in Fig. 4. Total B cells quantified as percentage of total live singlets. All other B cell populations quantified as percentages of total CD19+ B cells. Color scale denotes Spearman R. Stars denote significance. All shown data derived from HCT patients included in correlations, HCs excluded. All groupwise statistical comparisons by Kruskall-Wallis test with Dunn’s post hoc test comparing patient groups to healthy controls, unless otherwise specified. N = 21 (15 alloHCT, 5 HC) for 3A-B, 3I-J. N = 19 (14 alloHCT, 4 HC) for 3C-H. One alloHCT and one HC excluded due to technical problems with flow cytometry analysis. Where present, error bars indicate mean +/- SEM.

**
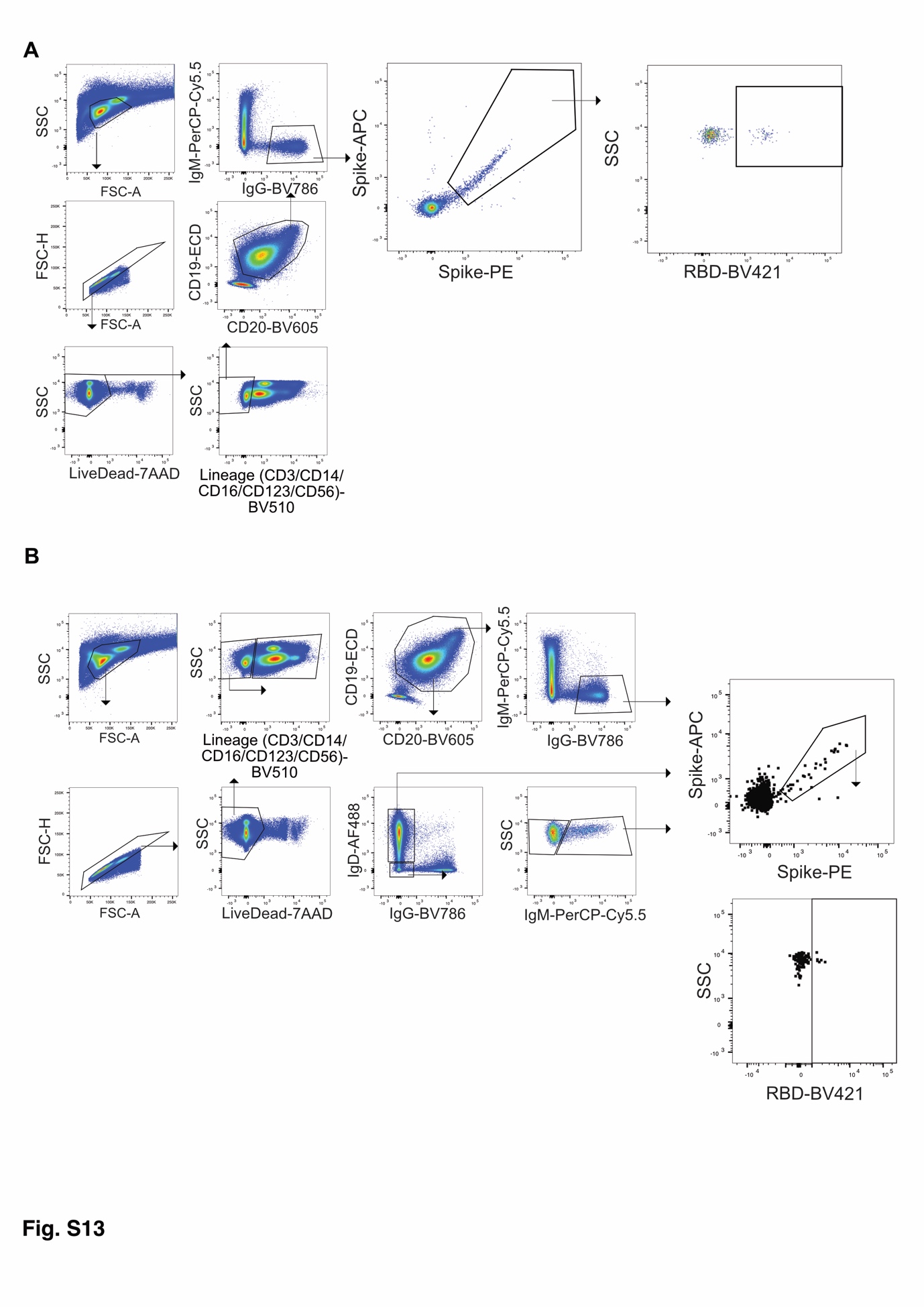
**

**Fig. S13:** Gating strategies used for sorting (**A**) and analysis (**B**) of Spike- and RBD-specific MBC.

**
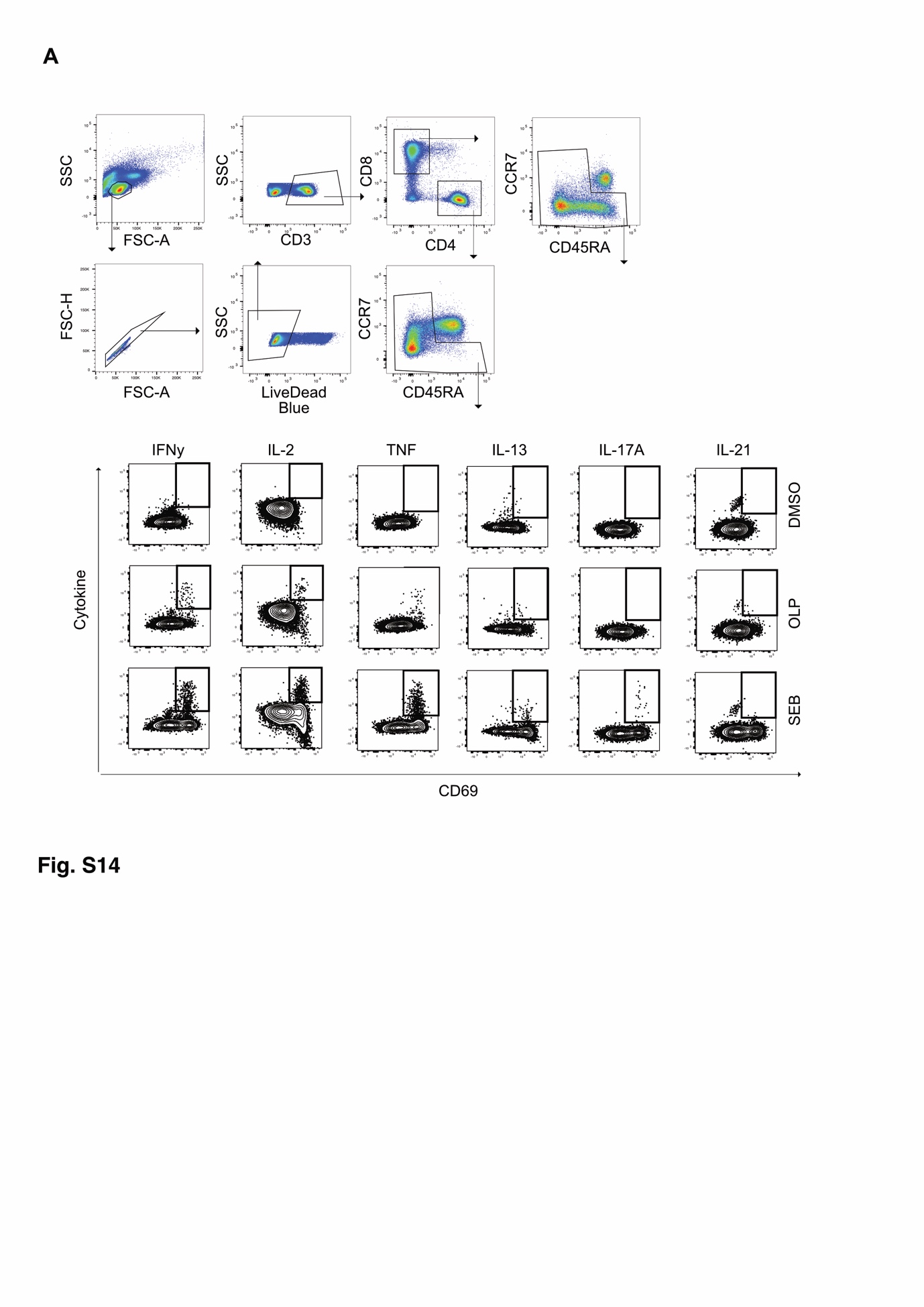
Fig. S14:** Gating strategies used for the identification of antigen-specific CD4 and CD8 T cells through *ex vivo* peptide stimulation and intracellular cytokine staining. Spike-OLP stimulated PBMC are shown in the second row of the lower panel. The same gating strategy was used for identification of CMV-specific T cells.

**
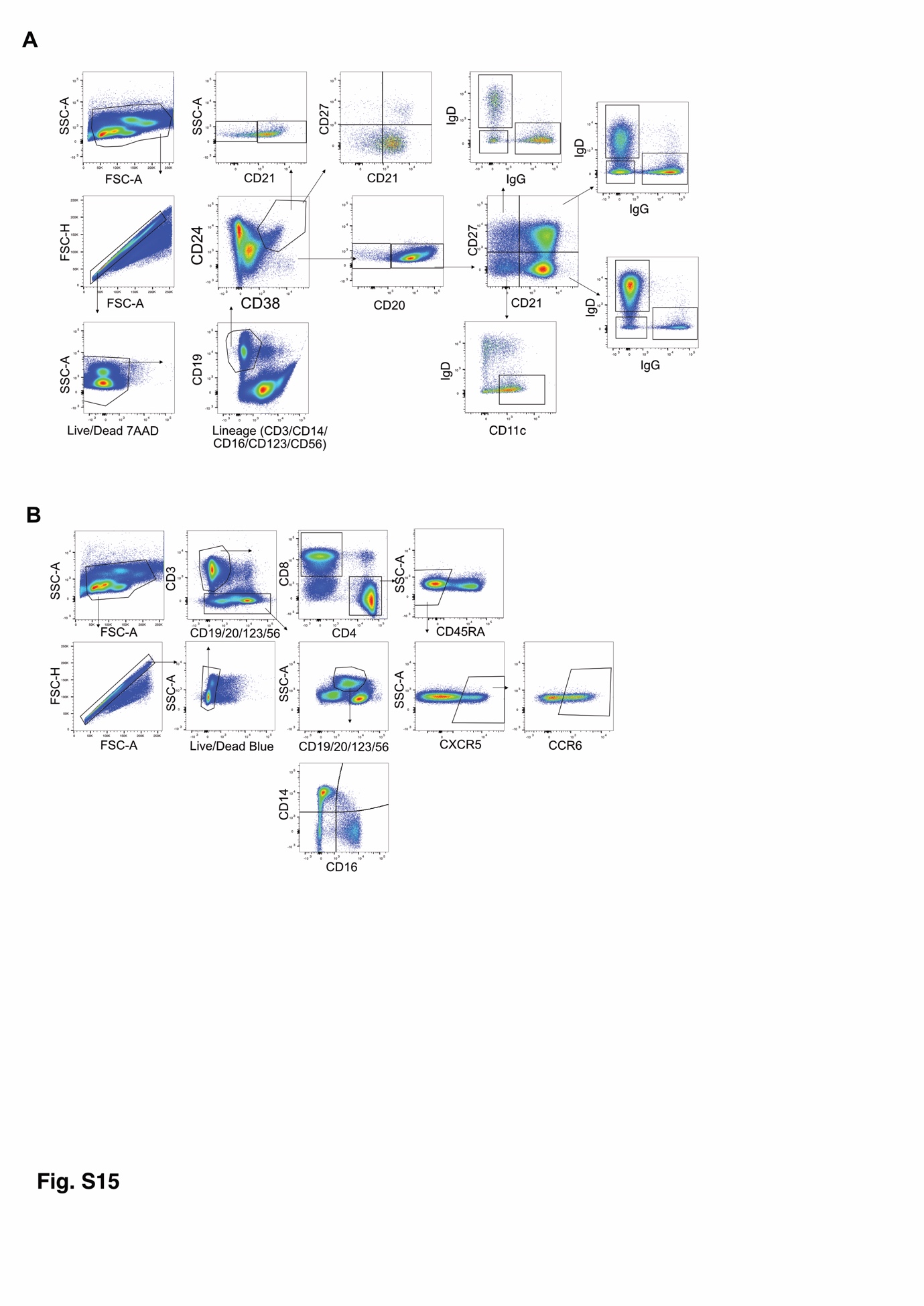
Fig. S15:** Gating strategies used for the identification of B cell **(A)** and T cell **(B)** subpopulations at Day 0.

**
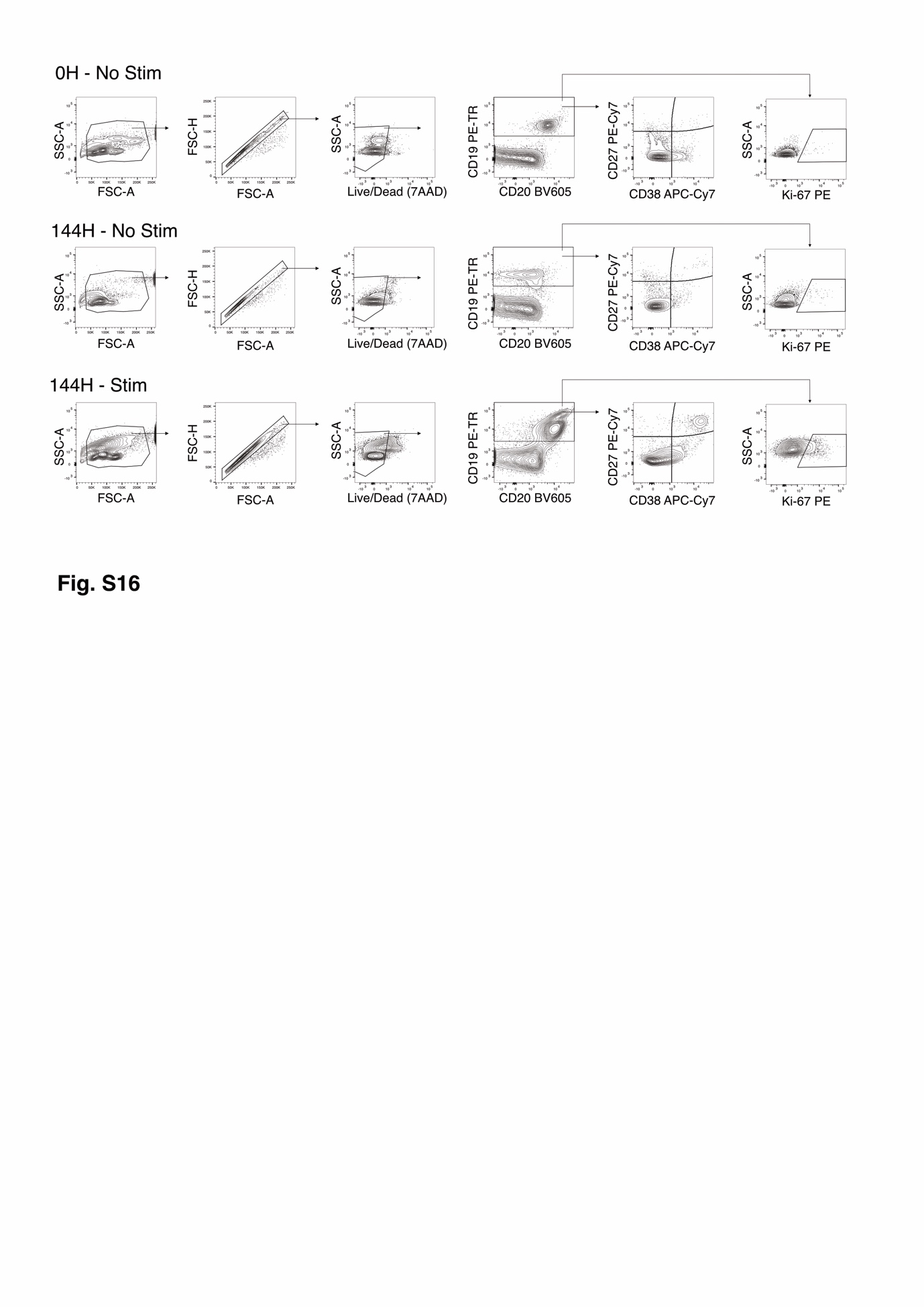
Fig. S16:** Gating strategy for the identification of ASCs and Ki-67+ proliferating B cells in stimulated PBMC cultures.
