# Supplementary material for: The impact of B‐cell reconstitution on mRNA vaccine responses in allogeneic stem cell transplant recipients
Source: Clin Transl Immunology. 2026 Feb 11;15(2):e70077. doi: 10.1002/cti2.70077 (PMC12892404; doi:10.1002/cti2.70077)
Supplement: Supplementary file 2 — Supplementary table 1 [file CTI2-15-e70077-s002.pdf]

Table S1: Cohort characteristics for participant subsets reported for key datasets/figures

Key data types (Figure reference)		D35 Binding titers (Fig 1B-C)		D35 VNT (Fig 1E)		D35 Binding avidity (Fig 1G)		D35 IgG/M Repertoire Sequencing (Fig 2)		D35 B cell Spike probing (Fig 3 B-D)		D35 S+ BCR sequencing Fig 3 E-F, S5		D35 S+ BCR repertoire analysis (Fig.3H-J)		D0 B cell phenotyping (Fig 4A-J, S12A-B)		D0 cTFH (Fig 4L)		ASC differentiation (Fig 5C-D, S12C-D)		IgG/IL-10 production (Fig 5I-L, S12E-F)		D35 S+ T cells (Fig. S4)		D0 T cells/innate (Fig. S10)	
**Type of treatment**	Allo HCT	77		29		72		16		35		27		15		35		23		14		15		20		23	
	CAR T	3		0		0		0		0		0		0		2**		0		0		0		2		2	
**Time from transplantation**	< 12 months	21	27%	12	41%	19	26%	7	44%	13	37%	10	43%	6	67%	14	40%	7	30%	5	36%	5	33%	8	40%	7	28%
	> 12 months	56	73%	17	59%	53	74%	9	56%	22	62%	17	57%	9	43%	21	60%	16	70%	9	64%	10	66%	12	60%	16	64%
**Sex**																											
	Male	43	54%	12	41%	38	53%	6	38%	16	46%	13	48%	6	40%	16	16%	9	70%	8	57%	8	53%	10	45%	11	44%
	Female	37	46%	17	59%	34	47%	10	63%	19	54%	14	52%	9	60%	19	27%	14	83%	6	43%	7	47%	13	59%	14	56%
**Age**	Median; min - max	59; 19 - 74		54; 20 - 74		59; 19 - 74		51.5; 20-74		58; 20 - 74		58; 20 - 74		52; 20 - 74		52; 20 - 74		54; 29 - 74		54; 30 - 73		54; 30- 73		53,5; 20-73		54; 29-74	
**Diagnosis**	AML	21		10		20		5		9		7		4		10		5		3		4		5		4	
	ALL	10		4		9		3		5		5		3		5		4		4		4		2		5	
	MDS	19		5		18		4		9		7		4		8		5		3		3		5		5	
	MPN	4		1		4		0		1		1		0		1		1		0		0		1		1	
	Lymphoma	17		7		13		3		10		6		3		12		7		4		4		8		9	
	Myeloma	3		1		2		1		1		1		1		1		1		0		0		1		1	
	Non-malignant	5		1		5		0		0		0		0		0		0		0		0		0		0	
	CML	1		0		1		0		0		0		0		0		0		0		0		0		0	
**Donor type**	HLA-identical sibling	22		8		19		5		11		7		5		11		7		4		4		6		7	
	Haploidentical family	3		1		3		1		2		2		1		2		1		2		2		2		1	
	Unrelated donor	52		20		50		10		22		18		9		20		15		8		9		12		13	
	Autologous / CAR T	3						0								2				0		0		2		2	
**Stem cell source**	Bone marrow	4		2		4		2		3		3		2		3		1		2		2		3		1	
	Peripheral blood	73		27		68		14		22		24		13		32		22		12		13		17		22	
**Conditining intensity**	Myeloablative	13		8		13		6		8		7		5		9		6		5		6		7		6	
	Reduced intensity	64		21		59		10		27		20		10		26		17		9		9		13		17	
**T-cell depletion**	ATG	58		21		55		10		14		18		9		24		17		8		9		5		17	
	Post-Cy	3		1		3		1		2		2		1		2		1		2		2		2		1	
	None	16		7		14		5		9		7		5		9		5		4		4		13		5	
**Ongoing Immunosuppression**	Yes	22	29%	10	34%	18	25%	7	44%	14	41%	10	37%	7	47%	14	38%	10	43%	6	43%	6	40%	9	69%	10	40%
	No	55	71%	19	66%	54	75%	9	56%	21	59%	17	63%	8	53%	21	62%	13	57%	8	57%	9	60%	13	31%	15	60%
**Prevaccination seropositive**		7*		1		6*		1		0		0		0		0		0		0		0		0		0	
**Covid-19 before day +35**		2		1		2		0		0		0		0		0		0		0		0		0		0	

*** One both seropositive and PCR positive**

**** CAR-T only in Fig. 4A**

**Healthy controls**																											
**N**		14		19		14		4		12		12		4		7		6		4		5		0		6	
**Sex**																											
	Male	7	50%	12	63%	7	50%	3	75%	8	67%	8	67%	3	75%	4	57%	4	67%	3	75%	4	80%			4	67%
	Female	7	50%	7	37%	7	50%	1	25%	4	33%	4	33%	1	25%	3	43%	2	33%	1	25%	1	20%			2	33%
**Age**	Median; min - max	67; 26 - 79		52; 22 - 76		67; 26 - 79		41; 22-52		52; 22 - 69		52; 22 - 69		41; 22-52		54; 22 - 69		53; 22 - 69		41; 22 - 69		52; 22- 69				53; 22-69	
