# Supplementary material for: The impact of B‐cell reconstitution on mRNA vaccine responses in allogeneic stem cell transplant recipients
Source: Clin Transl Immunology. 2026 Feb 11;15(2):e70077. doi: 10.1002/cti2.70077 (PMC12892404; doi:10.1002/cti2.70077)
Supplement: Supplementary file 3 — Supplementary table 2 [file CTI2-15-e70077-s003.pdf]

## Table 2: Bulk BCR sequencing information and lineage tracing per group

| *Group* | *Isotype* | *Antigen-specific clones** | *Antigen-specific clone size** | *Total BCR reads** | *Good quality** BCRs** | *Total clones** |
|---|---|---|---|---|---|---|
| HC | IgG | 29 (24.2-32.5) | 7056 (4819.2-8684.8) | 994893.0 (985024.0-1033630.0) | 948798.0 (939588.8-984376.8) | 4272 (4154.2-4385.8) |
| early_GvHD+ | IgG | 22 (21.5-22.5) | 1709 (1062.5-2355.5) | 1026630.0 (1023959.0-1626043.5) | 976667.0 (975561.5-1551139.5) | 5292 (5040.5-6098.0) |
| early_GvHD- | IgG | 25 (20.0-29.2) | 1716.5 (505.5-3077.2) | 796449.5 (697314.5-837972.8) | 760897.0 (663428.2-798028.0) | 4352 (3629.8-4790.0) |
| late_GvHD+ | IgG | 29 (25.0-58.0) | 3232 (885.0-27185.0) | 1121756.0 (1044544.0-1171814.0) | 1073768.0 (998823.0-1113684.0) | 5282 (4968.0-5469.0) |
| late_GvHD- | IgG | 57 (49.5-70.2) | 9633 (7311.5-17236.2) | 891985.5 (779357.2-1154774.8) | 847853.0 (737262.0-1099338.0) | 5472 (4933.0-5997.2) |
| HC | IgM | 14.5 (13.2-16.2) | 132.5 (119.5-155.2) | 318102.5 (291330.5-349956.0) | 293085.5 (268891.0-322790.0) | 4767.5 (4529.2-4910.2) |
| early_GvHD+ | IgM | 19.5 (16.2-22.8) | 173 (160.5-185.5) | 382011.0 (367633.0-453798.0) | 357106.0 (340829.0-421849.5) | 5970 (5187.0-6469.0) |
| early_GvHD- | IgM | 14.5 (13.5-15.5) | 159.5 (145.0-182.2) | 350190.0 (337359.2-364214.2) | 324140.0 (308876.2-340005.5) | 5292 (4638.8-5969.2) |
| late_GvHD+ | IgM | 15 (14.0-17.0) | 173 (164.0-228.0) | 370640.0 (364057.0-378895.0) | 340669.0 (334733.0-349372.0) | 4575 (4541.0-5734.0) |
| late_GvHD- | IgM | 28.5 (23.0-31.2) | 280.5 (245.0-299.2) | 463913.0 (420875.2-485966.8) | 428190.0 (387181.8-451357.0) | 7010 (6052.2-7608.0) |

* All values are expressed as median (interquartile range, Q1–Q3)

** Good quality includes sequences with no stop codon, good V-J genes assignment, and detected HCDR3.
